# Phage tailspike modularity and horizontal gene transfer reveals specificity towards *E. coli* O-antigen serogroups

**DOI:** 10.1186/s12985-023-02138-4

**Published:** 2023-08-07

**Authors:** Célia Pas, Agnieszka Latka, Lars Fieseler, Yves Briers

**Affiliations:** 1 Department of Biotechnology, Ghent University, Valentin Vaerwyckweg 1, 9000 Ghent, Belgium; 2 Department of Pathogen Biology and Immunology, University of Wroclaw, Przybyszewskiego 63, 51-148 Wrocław, Poland; 3 Centre for Food Safety and Quality Management, ZHAW School of Life Sciences and Facility Management, Einsiedlerstrasse 31, 8820 Wädenswil, Switzerland

**Keywords:** Shiga toxin-producing *Escherichia coli* (STEC), Tailspike, Receptor-binding protein (RBP), Horizontal gene transfer (HGT), Modular protein, Phage–host interaction

## Abstract

**Background:**

The interaction between bacteriophages and their hosts is intricate and highly specific. Receptor-binding proteins (RBPs) of phages such as tail fibers and tailspikes initiate the infection process. These RBPs bind to diverse outer membrane structures, including the O-antigen, which is a serogroup-specific sugar-based component of the outer lipopolysaccharide layer of Gram-negative bacteria. Among the most virulent *Escherichia coli* strains is the Shiga toxin-producing *E. coli* (STEC) pathotype dominated by a subset of O-antigen serogroups.

**Methods:**

Extensive phylogenetic and structural analyses were used to identify and validate specificity correlations between phage RBP subtypes and STEC O-antigen serogroups, relying on the principle of horizontal gene transfer as main driver for RBP evolution.

**Results:**

We identified O-antigen specific RBP subtypes for seven out of nine most prevalent STEC serogroups (O26, O45, O103, O104, O111, O145 and O157) and seven additional *E. coli* serogroups (O2, O8, O16, O18, 4s/O22, O77 and O78). Eight phage genera (*Gamaleya-, Justusliebig-, Kaguna-, Kayfuna-, Kutter-, Lederberg-, Nouzilly-* and *Uetakeviruses*) emerged for their high proportion of serogroup-specific RBPs. Additionally, we reveal sequence motifs in the RBP region, potentially serving as recombination hotspots between lytic phages.

**Conclusion:**

The results contribute to a better understanding of mosaicism of phage RBPs, but also demonstrate a method to identify and validate new RBP subtypes for current and future emerging serogroups.

**Supplementary Information:**

The online version contains supplementary material available at 10.1186/s12985-023-02138-4.

## Introduction

Bacteriophages (or phages) are viruses that infect bacteria. The phage–host relationship is specific and complex. Receptor-binding proteins (RBPs) of phages such as tail fibers and tailspikes are the first phage proteins interacting with the host, initiating the infection process. These proteins can specifically bind outer cell wall structures of bacteria such as capsular polysaccharides (CPS) [32, 57] or lipopolysaccharides (LPS) [21], (lipo)teichoic acids, outer membrane proteins, flagella, or pili [54]. Tail fibers generally adopt a fibrous shape and comprise a distal domain that binds the receptor, while tailspikes are typically shorter and contain an enzymatic domain that also degrades its receptor upon binding [12]. In this work we use the comprehensive term RBP due to inconsistently available information on the presence of such enzymatic activity. Whereas most phages encode a single or two RBPs, some polyvalent phages express multiple RBPs, forming a branched RBP structure. Each of these RBPs recognizes a different receptor, allowing the phage to infect multiple hosts [17, 31, 41, 45, 53].

Numerous phages infecting *Escherichia coli* encode RBPs targeting the outer layer of LPS, called the O-antigen. When this virulence factor is present on the *E. coli* outer cell wall, the structure is referred to as smooth LPS. A high O-antigen serogroup variability with 176 different structures has currently been described for smooth *E. coli* strains [37]. Among the most pathogenic *E. coli* strains is the Shiga toxin-producing *E. coli* (STEC) pathotype, being one of the main causes for gastrointestinal illnesses around the world. The prevalence of certain O-antigens associated to this pathotype varies across time as well as geographical location. The importance of STEC was recognized in 2015 by the Food and Agriculture Organization (FAO) of the United Nations and the World Health Organization (WHO). Serogroup O157 is the most prevailing serotype in the United States, although the share of non-O157 serogroups is continuing to grow. In 2020, more STEC cases were reported with serogroup O26 than cases carrying the O157 serogroup in Europe [13]. Additionally, isolated STEC outbreaks of new emerging serogroups can occur, like the O104 serogroup STEC outbreak in Germany in 2011 [28]. Other important non-O157 serogroups associated with human illness include O45, O91, O103, O104, O111, O145 and O146, with different prevalence in the USA versus Europe [15, 16].

Most tail fibers and tailspikes are homotrimeric, modular RBPs. They are generally composed of two domains: (1) an N-terminal anchor domain that functions as attachment domain of the RBP to the phage particle and (2) a C-terminal, receptor-binding domain (RBD) that is responsible for binding, and/or cleaving the host receptor. When this RBD has enzymatic activity, it generally displays a β-helical structure. The substrate-binding sites are located within the β-helix domain, either at the three interfaces between subunits (inter-subunit) as in the tailspike (TSP) of phage Sf6 and TSP1 and TSP2 of CBA120, or on the surface of each subunit (intra-subunit) such as in the TSPs of phages P22, Det7 and HK620 [7, 35, 40, 58, 64]. The RBD can optionally comprise small domains such as a chaperone, adhesin or carbohydrate binding domain. The C-terminal RBD is highly subjected to horizontal gene transfer (HGT) and is often exchanged both within and outside the phylogenetic borders of the phage genera, whereas the N-terminal anchor domain remains conserved within a phage genus [21, 31, 46]. Certain phages make use of sequence motifs to aid recombination, resulting in high mosaicism in the genome [2, 25]. Such potential motifs have also been identified within the RBP gene [56, 61].

This work demonstrates the O-antigen binding potential of RBPs of members from eight phage genera, namely the *Gamaleya-, Justusliebig-, Kaguna-, Kayfuna-, Kutter-, Lederberg-, Nouzilly-* and *Uetakeviruses*. We confirm that the selection of phages expressing RBPs of the same subtype recognizes hosts with the same serogroups and we predict the serogroup specificity of various RBPs in silico based on phylogenetic and structural clustering. Additionally, we identified RBD-surrounding DNA sequence motifs that are conserved in RBP genes across the lytic phage genera studied here.

## Materials and methods

### General methodology

**Fig. 1:**
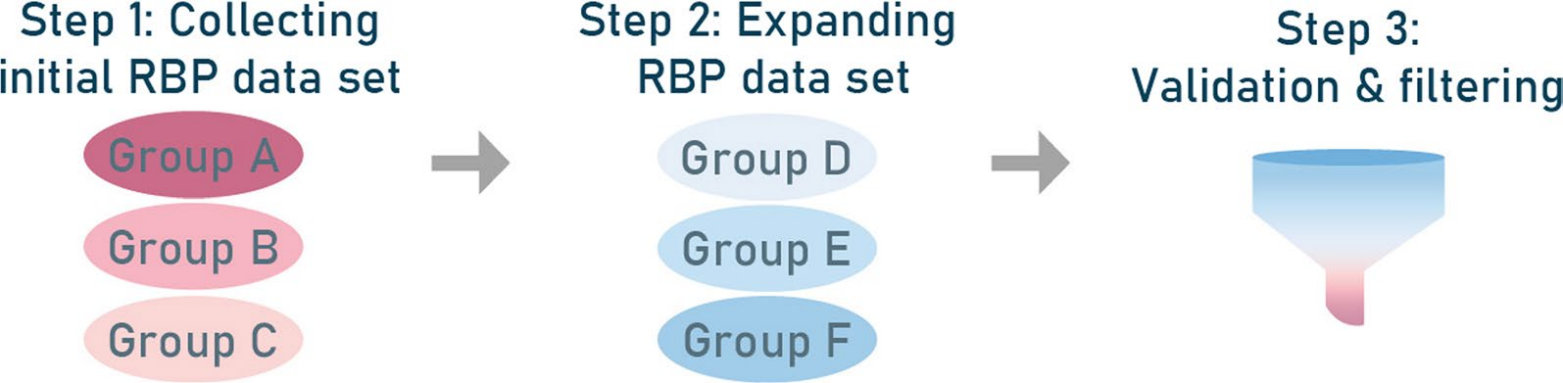
Overview of the methodological pipeline that was used to identify and validate RBPs. Step 1 entailed the collection of an initial RBP data set (groups A, B and C), which was further expanded in step 2 (groups D, E and F). In the final step 3, different RBP subtypes were validated and strictly filtered for a high probability of *E. coli* O-antigen specificity

A methodological pipeline was developed to identify and validate putative O-antigen specific RBPs. Generally, the pipeline consisted of three steps (Fig. 1), covering the set-up of an initial RBP data set (Step 1), the expansion to an expanded RBP data set (Step 2) followed by the validation and filtering of both data sets to obtain a final curated data set of O-antigen serogroup-specific RBPs (Step 3).

In Step 1 relevant RBPs were collected for the initial data set based on experimentally validated O-antigen specific RBPs at the RBP (group A) or indirectly at the phage (group B) level. The initial data set was further supplemented through the identification of horizontal transfer events of the C-terminal domains of group A and B RBPs across phage genera, resulting in additional group C RBPs. Through phylogenetic clustering of group A, B and C RBPs, different RBP subtypes were established with a corresponding O-antigen serogroup.

In Step 2, additional putative O-antigen specific RBPs were identified based on a dominant link between prophage RBP specificity and the O-antigen of the prophage host (group D), based on genera that are enriched for such O-antigen specific RBPs (group E) and the identification of additional horizontal transfer events of the C-terminal domains from group A-E RBPs (group F).

In Step 3, a quality control was performed based on validation and filtering to retain only those RBP subtypes that have a high probability to target a single O-antigen serogroup.

#### Step 1: collecting initial RBP data set

A pool of phages with experimentally verified *E. coli* serogroup specificity was collected from literature using search query ‘phage tailspike O-antigen Shiga toxin-producing *E. coli*’ on Google scholar (accessed November 15, 2022). Phage RBPs with experimentally confirmed serogroup specificity at the RBP level were labeled as group A. Some phages encode multiple RBPs that form a branched RBP structure. When multiple RBPs were detected, the separate RBPs were identified and numbered according to the RBP order in their reference phages (phages CBA120 and G7C). The RBPs of phages that are serogroup-specific, confirmed at the phage level through host range testing, were labeled as group B. For group B, only phages with a single RBP (confirmed with MAUVE progressive alignment [11] against closely related phages from the same genus) were selected, as it is assumed that the RBP is responsible for the interaction with the O-antigen of the host.

To specifically focus on the HGT of the RBDs from group A and B RBPs, a tentative N-terminal anchor cutoff of 150 aa was chosen [34, 49, 50]. BLASTp (searched in *Caudoviricetes* (taxid: 2,731,619)) was performed with the tentative C-terminal RBD sequences beyond this 150 aa cutoff. An aa identity of ≥ 30% identity and ≥ 60% coverage was chosen to select for HGT events. This choice was made based on our previous analyses and the basics of homology modeling [41, 42, 65]. When an RBP was identified with similarity to the query RBP, and the phage encoding the RBP had at least one known host belonging to the same serogroup as the host of the query phage, the phage and its RBP were withheld. Only taxonomically classified *Escherichia* and *Enterobacteria* phages with annotated RBPs were retained in this search. Unverified sequences or genomes with misannotated RBP coding sequences (CDS) were discarded. These newly identified RBPs belong to group C. When multiple strains of the same serogroup encoded a highly similar RBP (≥ 80% aa identity), only one RBP was withheld to avoid RBP redundancy. Next, the RBPs from group A, B and C with known serogroup specificity were classified into RBP subtypes (≥ 30% identity and ≥ 60% coverage based on BLASTp).

#### Step 2: expanding the data set with potential O-antigen-specific RBPs

The initial data set was expanded with potential O-antigen-specific RBPs from prophages (group D), based on taxonomy (group E) and the identification of HGT events (group F). For group D, prophages integrated in strains belonging to the foremost important serogroups prevalent among STEC strains, specifically O26, O45, O91, O103, O104, O111, O145, O146 and O157 were selected. First, to select for *E. coli* genomes having the desired serogroups, we used BLASTn [48] with as query sequence the O-antigen biosynthesis gene cluster of the respective serogroups (DQ196413.1, AY771223.1, AY035396.1, AY532664.1, AF361371.1, AF078736.1, AY863412.1, DQ465249.1, AF061251.1). The withheld strains were subsequently screened for *Lederberg-* and *Uetakeviruses* prophages using tBLASTn. The query sequences used are the first 205 and 124 aa of the RBPs of the respective phages phiV10 (*Uetakevirus*, YP_512279.1) and HK620 (*Lederbergvirus*, NC_002730.1), as described in the paragraph ‘Delineation Anchor-RBD’. When an *E. coli* strain with a predicted *Uetake-* or *Lederbergvirus* prophage was found, the serogroup of the strain was further confirmed using SerotypeFinder [24] with ≥ 95% aa identity. Next, the Prophage Hunter tool [55] was used to extract the active (Prophage Hunter score of > 0.8) prophage genome sequences. An exception was made for some *Lederbergviruses* due to their highly variable genome. When no active prophages were found, prophages with a score between 0.5 and 0.8 (labeled as ‘ambiguous’) were also used. Prophage genomes were annotated using the KBase platform [1] with the RAST annotation tool [4]. When multiple strains of the same serogroup encoded a highly similar RBP (≥ 80% aa identity), only the prophage with the highest Prophage Hunter score was withheld to avoid RBP redundancy.

For group E, all verified genomes of phages belonging to the seven genera described in the initial data set (*Gamaleya-, Kaguna-, Kayfuna-, Kutter-, Lederberg-, Nouzilly-* and *Uetakeviruses*) were collected from the NCBI database (accessed January 16, 2023). Their RBPs were selected and added to the data set. Phages with smooth *E. coli* strains, unspecified hosts or *E. coli* as host organism were withheld but phages with commonly used rough *E. coli* host strains, namely strains 58, AG1, B, BL21, C, C600, DH1, DH5α, MG1655, W3110 and W945 [22, 30] as host organism were discarded. To avoid RBP redundancy, only one phage representative was chosen for each RBP subtype within the same phage genus (≥ 30% aa identity, ≥ 60% coverage over the tentative RBD).

For group F, HGT events of the RBP subtypes from group A–E were identified across phage genera using the tBLASTn tool (≥ 30% aa identity and ≥ 60% coverage over the tentative RBDs) within *Caudoviricetes* (taxid: 2,731,619) and *Enterobacteriaceae* (taxid: 543). *Caudoviricetes* hits were added to the data set, but RBPs from unclassified phages were discarded. When a RBP was identified in an additional phage genus, phages were again collected from these genera as described for group E. If BLAST hits were found in *E. coli* strains, the pipeline of collecting prophage RBPs (group D, SerotypeFinder, Prophage Hunter, RAST annotation) was repeated to obtain the RBPs of the phages. In line with group D and E, a single RBP representative was chosen for every RBP subtype within a phage genus.

#### Step 3: serogroup specificity validation and filtering

Upon collection of the complete data set (groups A–F; n = 136), all RBPs could be clustered in 64 different RBP subtypes. Subsequently, the serogroup specificity was validated per RBP subtype. For each RBP within the RBP subtype, the serogroup of the host strain of the phage encoding the RBP or the serogroup host strain in which the prophage was integrated was identified using SerotypeFinder [24]. Next to the 136 RBPs, all host serogroup information of the RBP doubles (RBPs of the same RBP subtype within a single phage genus) were analyzed. The following criteria applied to assign a serogroup to a particular subtype: (i if a group A or B member (experimentally confirmed at the RBP level or at the phage level was present in the RBP subtype, the O-antigen serogroup of this member was assigned to the whole RBP subtype; (ii for all other RBP subtypes, at least 90% of the serogroups must be identical (with a minimum of two confirmed serogroups. To RBP subtypes that did not meet these criteria, no O-antigen serogroup was assigned. To obtain the final data set, we withheld those phage genera that have at least two O-antigen-specific RBPs.

### Genome and RBP phylogeny

Phage whole genome alignment was performed by VICTOR phylogeny, using the genome-BLAST distance phylogeny (GBDP) method [39]. The delineation of anchor and RBD were chosen as described in the paragraph** ‘**Delineation Anchor-RBD’. MAFFT MSA (G-INS-1) uses the neighbor-joining method and was utilized to construct accurate phylogenetic tree data of the collected RBPs and their respective anchors and RBDs [27]. All phylogenetic trees were visualized using Interactive Tree Of Life (iTOL) v5 [36] and the layout was edited using Adobe Illustrator version 25.4.1.

### RBP region visualization

The Clinker genome visualization tool [19] was used to illustrate homology between CDSs in the RBP region. For this purpose, circular genomes were linearized using SnapGene software (www.snapgene.com). All figures were polished using Adobe Illustrator version 25.4.1 and Adobe InDesign version 16.4.3.

### Percentage identity matrix

Amino acid and DNA sequences of the RBPs were aligned using MUSCLE multiple sequence alignment (MSA) [14]. For the identification of motif sequences, RBPs showing DNA sequence homology were selected and re-aligned. The MSA was visualized using SnapGene software (www.snapgene.com) and the resulting percentage identity matrix was visualized as a heat-map using python, version 3.10.4 [62] and matplotlib, version 3.6.3 [23].

### Delineation anchor-RBD

HGT events of RBP sequences were analyzed in depth using MUSCLE multiple sequence alignment (MSA) when RBP sequences within a single phage genus were compared [14]. Domain delineations were manually curated based on the investigation of HGT events within the RBP coding sequence by locating flexible linker domains in the predicted tertiary protein structures of the RBP monomers and by previously confirmed experimental data of well-investigated phage genus member RBPs. Chosen delineations can be found in Additional file 1: Table S1.

### RBP structure prediction

AlphaFold2 (v2.1.1; multimer, maximum recycles = 12) was used on the HPC-UGent to predict the homotrimeric quaternary structures of the RBPs [26]. When quaternary structure predictions failed, the full RBP sequence was split in the N-terminal anchor domain and C-terminal RBD and separate predictions were made. For coloring, the anchor domain comprised the N-terminal phage tail-binding domain and if present, the separating α-helix. The RBD was chosen to comprise all domains downstream of this anchor domain. Structures were further processed and root-mean-square deviations (RMSDs) were calculated using the PyMol Molecular Graphics System, version 2.5.2 [52], Blender, version 2.93.3 [10] and Adobe InDesign version 16.4.3.

## Results

### Modular RBP evolution is driven by horizontal gene transfer across genera

To investigate the modular evolution of O-antigen serogroup-specific RBPs from phages infecting prevalent STEC serogroups, we first assembled an initial data set of phage RBPs (Step 1; Fig. 1, Additional file 1: Table S1), using three different sources:

1. *Escherichia coli* phage RBPs with experimentally verified O-antigen serogroup specificity, confirmed at the RBP level (group A; n = 8; RBPs of phages CBA120 (RBP2, RBP3 and RBP4), EP75 (RBP1), G7C (RBP2), HK620, LB226692_Prophage and phiV10). Phage CBA120 belonging to the *Kuttervirus* genus, encodes four separate RBPs. RBP2, RBP3 and RBP4 have been experimentally verified to cleave the O157, O77 and O78 antigen, respectively (TSP2, TSP3 and TSP4 [45]). Phages of the *Gamaleyavirus* genus encode two O-antigen specific RBPs, including phage G7C of which the second RBP (RBP2) was demonstrated to cleave the 4s/O22 O-antigen (gp63.1, [46],
2. *Escherichia coli* phage RBPs with experimentally verified O-antigen serogroup specificity at the phage level when the respective phage encodes only a single RBP (group B; n = 4; RBPs of phages CLB_P1, Ro103C3Iw, Ro145c2YLVW and Ro45lw);
3. *Escherichia coli* phage RBPs identified by HGT across phages. The inclusion criteria are (1) that the RBP belongs to the same RBP subtype (≥ 30% aa identity across the RBD) to one of the experimentally validated O-antigen serogroup-specific RBPs from group A and/or B, and (2) that the respective phage infects a host with the same serogroup (group C; n = 5; RBPs of phages ESCO41, Penshu1, PhAPEC7 (RBP2), phiWAO78-1 and TL-2011b).

This initial selection comprised 17 phage RBPs encoded by seven different phage genera (*Gamaleya-, Kaguna-, Kayfuna-, Kutter-, Lederberg-, Nouzilly-* and *Uetakeviruses*) and belonging to six distinct RBP subtypes, one for each of six serogroups (O18, 4s/O22, O78, O103, O104 and O157). Group A RBPs have the highest confidence level in terms of specificity prediction since they are directly experimentally validated, whereas the confidence level reduces for group B and further for group C RBPs since their predictions are increasingly based on indirect evidence.

**Fig. 2:**
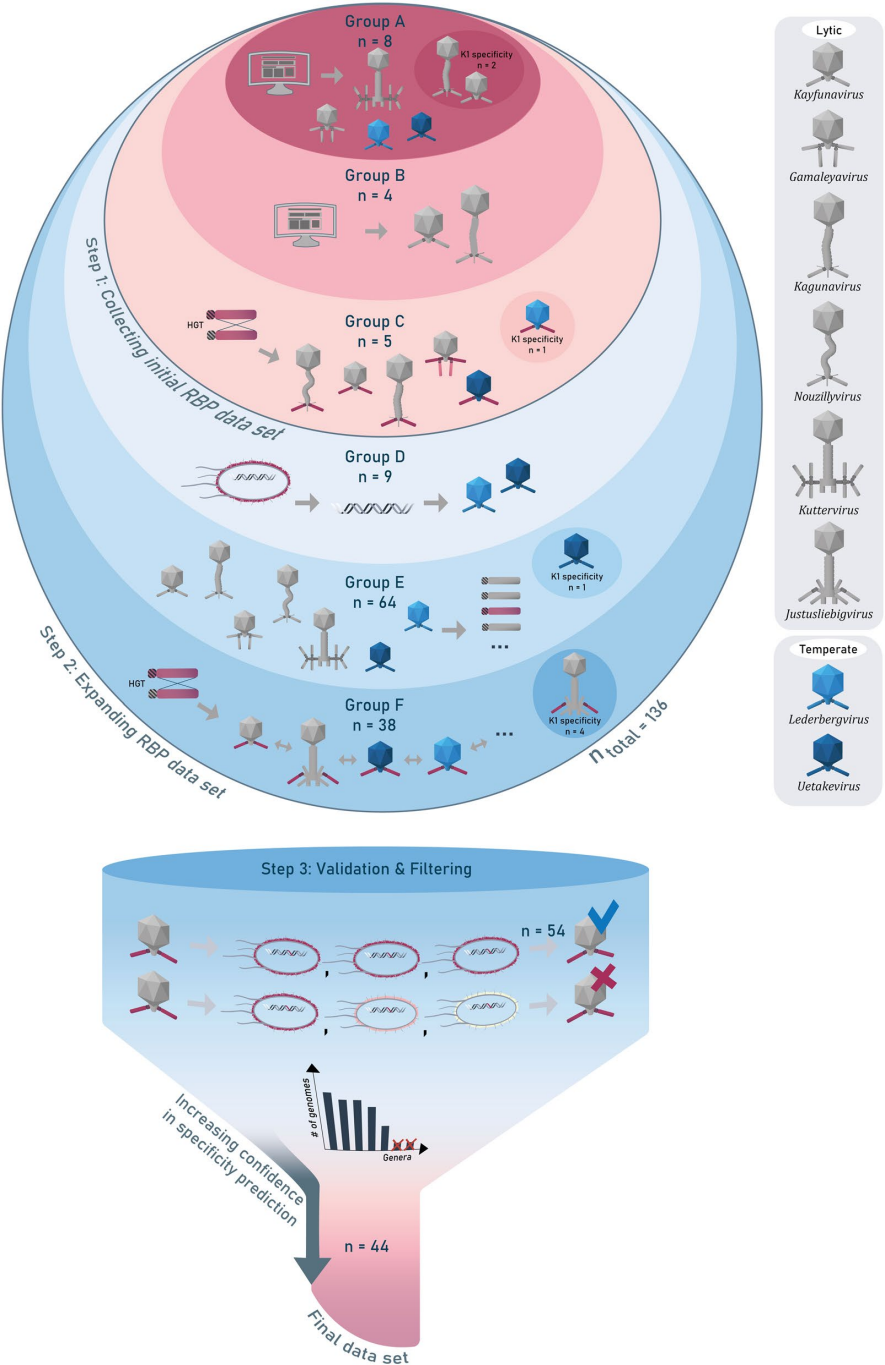
Overview methodology of this study. An initial (group A–C) and expanded (group A–F) data set was acquired using three steps (Fig. 1). In step 1, reference phages were collected from previously published work resulting in group A RBPs with serogroup specificity confirmed at the RBP level and group B RBPs derived from phages with a single RBP and experimentally confirmed serogroup specificity at the phage level. Using the principle of HGT, group C RBPs were identified, belonging to the same RBP subtype as the RBPs from group A and B and infecting the serogroup of the respective subtype. In step 2, the data set was expanded. RBPs from *Lederberg-* or *Uetakevirus* prophages of relevant STEC serogroups were selected in group D. Group E comprises a collection of all RBPs of *E. coli* phages within the seven genera obtained through group A–D. Group F uses the principle of HGT to identify all RBPs in the NCBI database that belong to the same RBP subtypes as those in group A–E. Note that only one RBP representative was withheld for each RBP subtype within a single phage genus to avoid RBP redundancy. K1-targeting RBPs were added throughout the pipeline as an outgroup. Finally, serogroup specificity of all RBP subtypes was validated in step 3, through a consistency analysis of the host serogroups of the phages containing a RBP from the same RBP subtype

To complete the initial data set, we have included three *E. coli* phage RBPs specific for the K1 capsule as an outgroup. RBP specificity for capsule serotypes and their evolution by HGT events has been well documented. *Kayfunavirus* K1F and *Kagunavirus* K1H, which have homologous (49% aa identity) and experimentally verified RBPs targeting capsule K1 [43, 51], are added to group A. A third phage phiv205-1 has undergone an apparent HGT event, encoding a RBP belonging to the K1-specific RBP subtype of phages K1F and K1H (63.1 and 55.6% aa identity, respectively), and infects an *E. coli* strain with capsular serotype K1 (added to group C, n = 1) (Fig. 2).

**Fig. 3:**
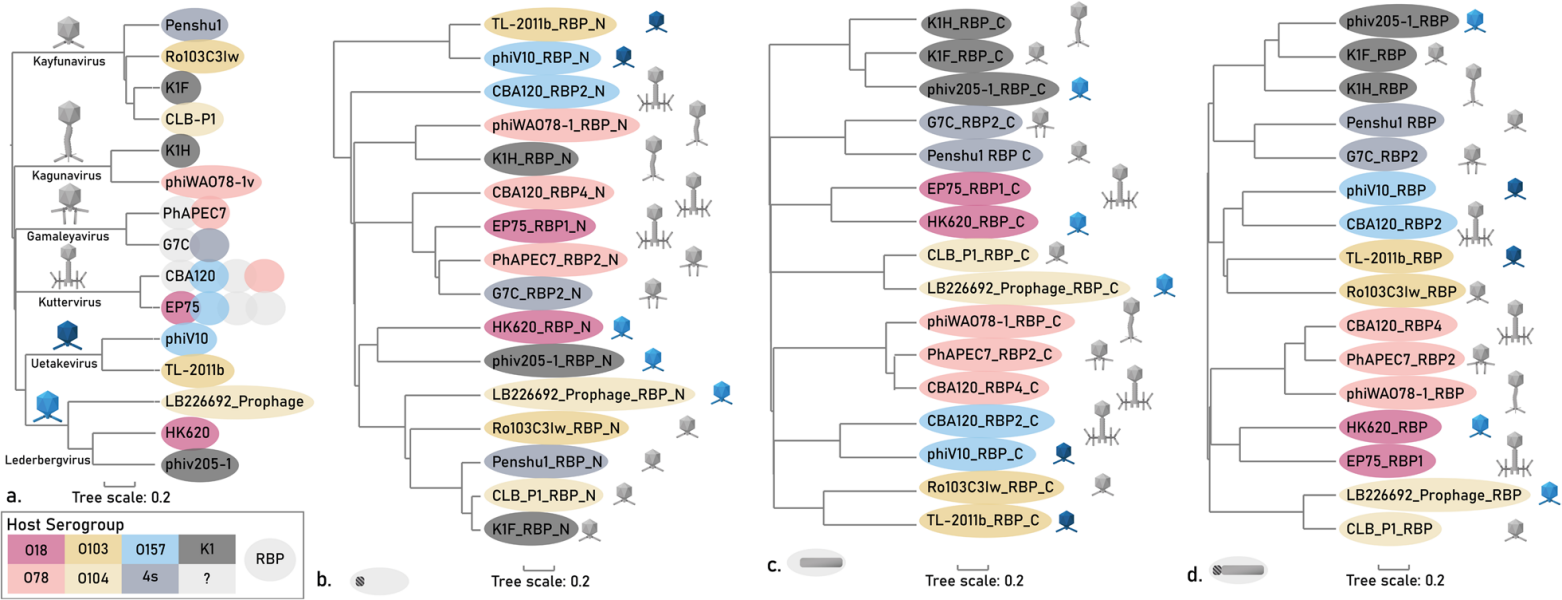
Phylogenetic trees of phages and their RBPs from the initial data set **a** Phylogenetic tree of the whole phage genomes using VICTOR phylogeny. Phylogenetic trees based on MAFFT G-INS-1 alignment of **b** the N-terminal anchor domains of the RBPs of interest, **c** the C-terminal RBD of the RBPs and **d** the complete RBP coding sequences. The color of the ellipses indicates the targeted O-antigen by the RBP, whereas the phage particle morphology indicates the phage taxonomic group as indicated in panel a. For phages encoding multiple RBPs, the RBPs were displayed according to gene order (e.g., second ellipse of CBA120 represents RBP2). RBPs of the same subtype and belonging to phages of the same genus were only shown once, except for RBP2 of phage EP75 in panel a. Note that four RBPs of the initial data set that are singletons are not included in the figure (RBP3 of phage CBA120 and the RBPs of phages ESCO41, Ro145c2YLVW and Ro45lw)

The initial data set comprises 20 RBPs. Separate phylogenetic trees were set up for the phage genomes of this initial data set and their RBPs (Fig. 3). The RBP sequences were delineated for their N-terminal structural anchor domain responsible for attachment to the phage particle, and their C-terminal RBD responsible for receptor recognition. Subsequently, phylogenetic trees were created for the N-terminal structural anchor domains and C-terminal RBDs of the RBPs separately. The phage genome and anchor domains cluster according to phage taxonomy (Fig. 3a, b), whereas the RBD of the RBP leads to a clustering according to the corresponding host serotype (Fig. 3c). Clustering according to host serotype was also visible when using the complete RBP coding sequence, which can be explained by the generally longer length of the RBD compared to the anchor domain (Fig. 3d). This excellent clustering within the initial data set demonstrates how HGT events of the RBD domains within and across phage genera have shaped the RBP and phage specificity, whereas the anchor domains are conserved within phage genera to enable attachment to the phage tail.

### An expanded pipeline to identify potential O-antigen specific RBPs from *E. coli* phages

The observation of a strict correlation of RBP subtypes within and across phage genera with O-antigen serogroup specificity in the initial data set encouraged us to expand the pipeline to identify more potential O-antigen-specific RBPs from *E. coli* phages and to map the broad diversity of RBP subtypes per serogroup. Therefore, as a second step in our pipeline, we expanded our initial data set with additional RBPs with a high potential to be serogroup-specific (Step 2; Fig. 2; Additional file 1: Table S1). Three different sources based on prophages (group D), taxonomic relationships (group E), and identified HGT events (group F) were used, inspired by different rationales that support O-antigen specificity of the respective RBPs. For the entire expanded data set, only one RBP representative was withheld for every RBP subtype within a phage genus to avoid RBP redundancy.

The first group (group D) of RBPs relies on the hypothesis that an integrated prophage usually encodes a RBP that allows to infect the bacterial strain in which it is integrated [5]. This hypothesis was only applied to prophages that have a single RBP to ensure that potential O-antigen specificity is assigned to the right RBP. We focused particularly on the temperate phage genera *Lederberg-* and *Uetakeviruses*. The latter are exemplary phage genera for experimentally validated O-antigen-specific RBPs, as identified in the initial data set (group A).

Following this principle, RBPs originating from *Uetakevirus* and/or *Lederbergvirus* prophages integrated in strains of the most prevailing STEC serogroups O26, O45, O91, O103, O104, O111, O145, O146 and O157 were identified (n = 9, group D). The prevalence of prophages was highly variable across the different serogroups (Additional file 2: Table S2), but strains from all serogroups contained at least one prophage belonging to one of the two genera, except for serogroup O91. While for O103 strains eight out of 32 (25%) encoded a prophage belonging to one of these genera, only one out of 39 screened O157 strains did (< 3%). For most strains, no more than one prophage of each genus was found within a single genome. After removing all redundant RBPs within a single genus and RBP subtype (≥ 30% aa identity over the tentative RBD), distinct *Uetakevirus* prophage RBPs were retrieved from strains with serogroups O26 (n = 1), O45 (n = 1), O103 (n = 2) and O146 (n = 1), and *Lederbergvirus* RBPs for serogroups O26 (n = 1), O103 (n = 1), O111 (n = 1) and O145 (n = 1).

The rationale for group E was based on the observation that all RBPs from group A-D belonged to only seven genera, *i.e.*, *Gamaleya-, Kaguna-, Kayfuna-, Kutter-, Lederberg-, Nouzilly-* and *Uetakeviruses*. Therefore, we reasoned that these genera may be enriched for O-antigen-specific RBPs, and we expanded the data set with all RBPs from these genera upon manual curation and filtering (n = 65).

Group F RBPs were added based on the identification of HGT events (n = 42). For this, we relied on the modularity principle of RBPs that retain conserved anchors for structural reasons, while swapping the RBD for specificity switches. Using tBLASTn searches with the previously identified tentative RBD subtypes (group A–E) as query, new RBPs linked via a HGT event were detected including in the previously unexplored genus *Justusliebigviruses*. This additional phage genus was then further mined as described for group E RBPs. Nine out of 42 RBPs in this group were obtained from intact prophages of *E. coli* strains.

### Validation of RBP subtypes relying on the conservation of serogroup specificity across HGTs

**Fig. 4:**
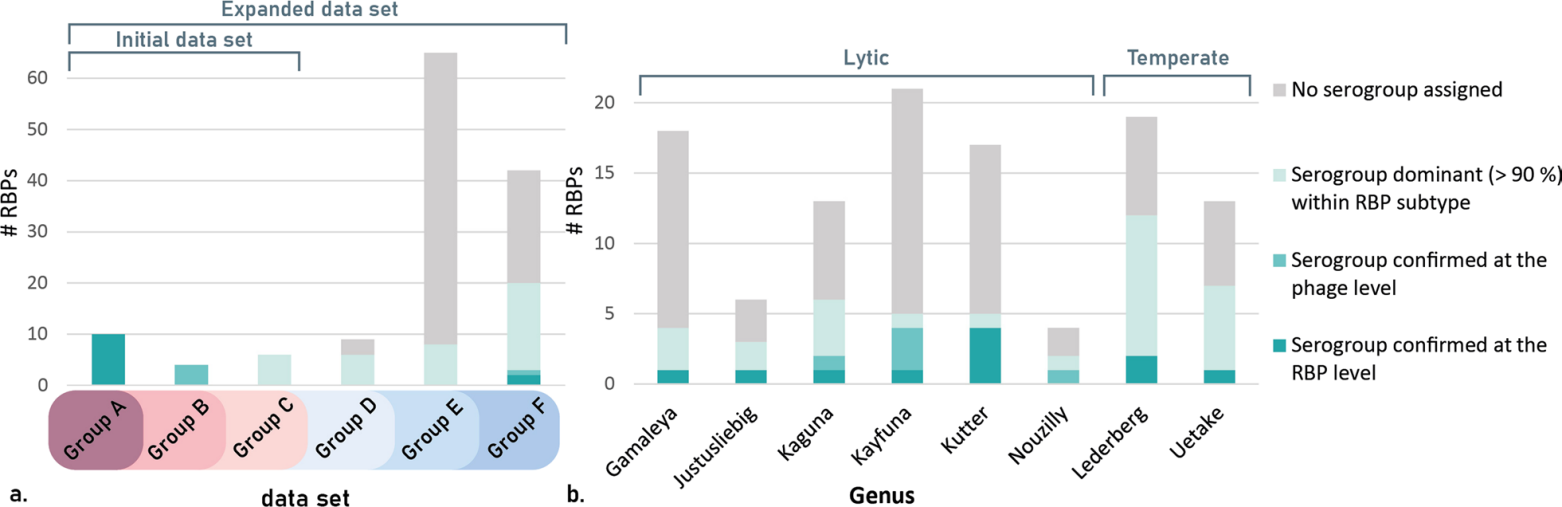
Validation of the expanded data set and filtering to the final data set. **a** The assignment of a serogroup per RBP is visualized per group of the initial (group A–C) and expanded (group A–F) data set. All stacked bars amount to the total number of RBPs that were collected in the expanded data set (n = 136). Serogroup specificity was assigned to RBPs (n = 54) based on experimental validation at the RBP level (dark green), at the phage level (green) or when at least 90% of the RBPs of a particular RBP subtype (with a minimum of two) have the same predicted serogroup (light green). All other RBPs (grey) were discarded since they were predicted to not be serogroup-specific or due to a lack of confidence. **b** Serogroup prediction of RBPs is shown per phage genus, limited to genera containing at least two serogroup-confirmed RBPs

At this stage, the expanded data set (group A–F) comprised 136 RBPs (Additional file 1: Table S1), which were subsequently subjected to a final validation step to select for O-antigen serogroup RBPs only (Step 3; Fig. 2). First, all RBPs were clustered (based on ≥ 30% aa identity over the tentative RBD), resulting in 64 different RBP subtypes. Secondly, the serogroup of the host strain of the phage encoding a RBP or the serogroup of the host strain in which the prophage was integrated was listed for each RBP of the RBP subtype cluster. The following criteria were then applied to assign a serogroup to each particular RBP subtype: (i) if a group A or B member (experimentally confirmed at the RBP level or at the phage level) was present in the RBP subtype, the O-antigen serogroup of this member was assigned to the whole RBP subtype; (ii) for all other RBP subtypes, at least 90% of the host strain serogroups must be identical (with a minimum of two RBPs). Importantly, we should note that all RBP subtypes that fulfill criterion (i), also fulfill criterion (ii). Based on these criteria, an O-antigen serogroup could be assigned to 15 RBP subtypes comprising 54 RBPs in total. The remaining RBP subtypes either showed inconsistency in host serogroups (25 RBP subtypes comprising 49 RBPs) or no serogroup could be assigned due to a lack of data (22 RBP subtypes comprising 31 RBPs) (Fig. 4a). More RBPs of the final, validated data set originate from group F than group E, indicating that the method of using HGT to identify new O-antigen specific RBPs was more efficient than the method of extracting RBPs from phages belonging to the same genera.

### In-depth analysis of HGT of RBPs from selected phage genera

**Fig. 5:**
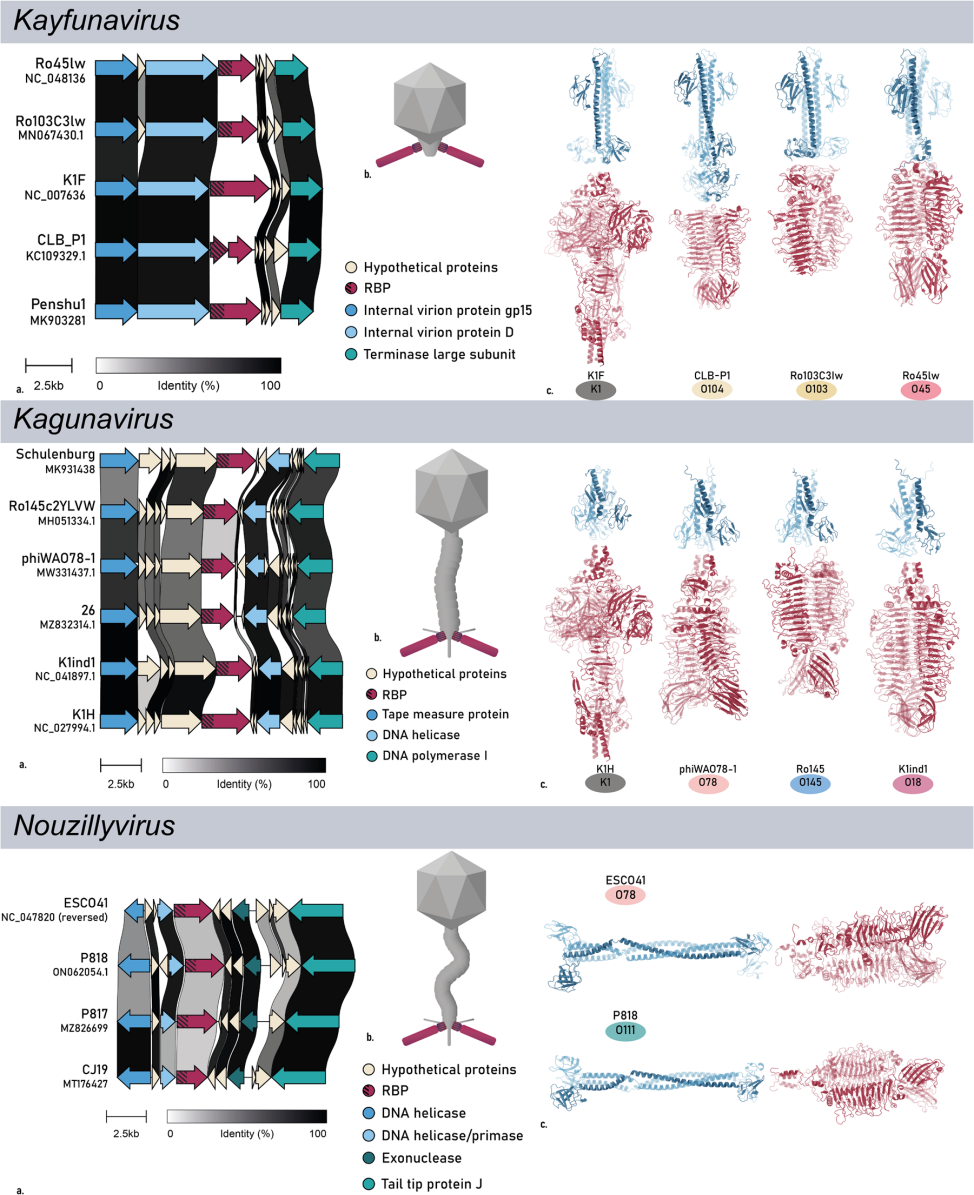

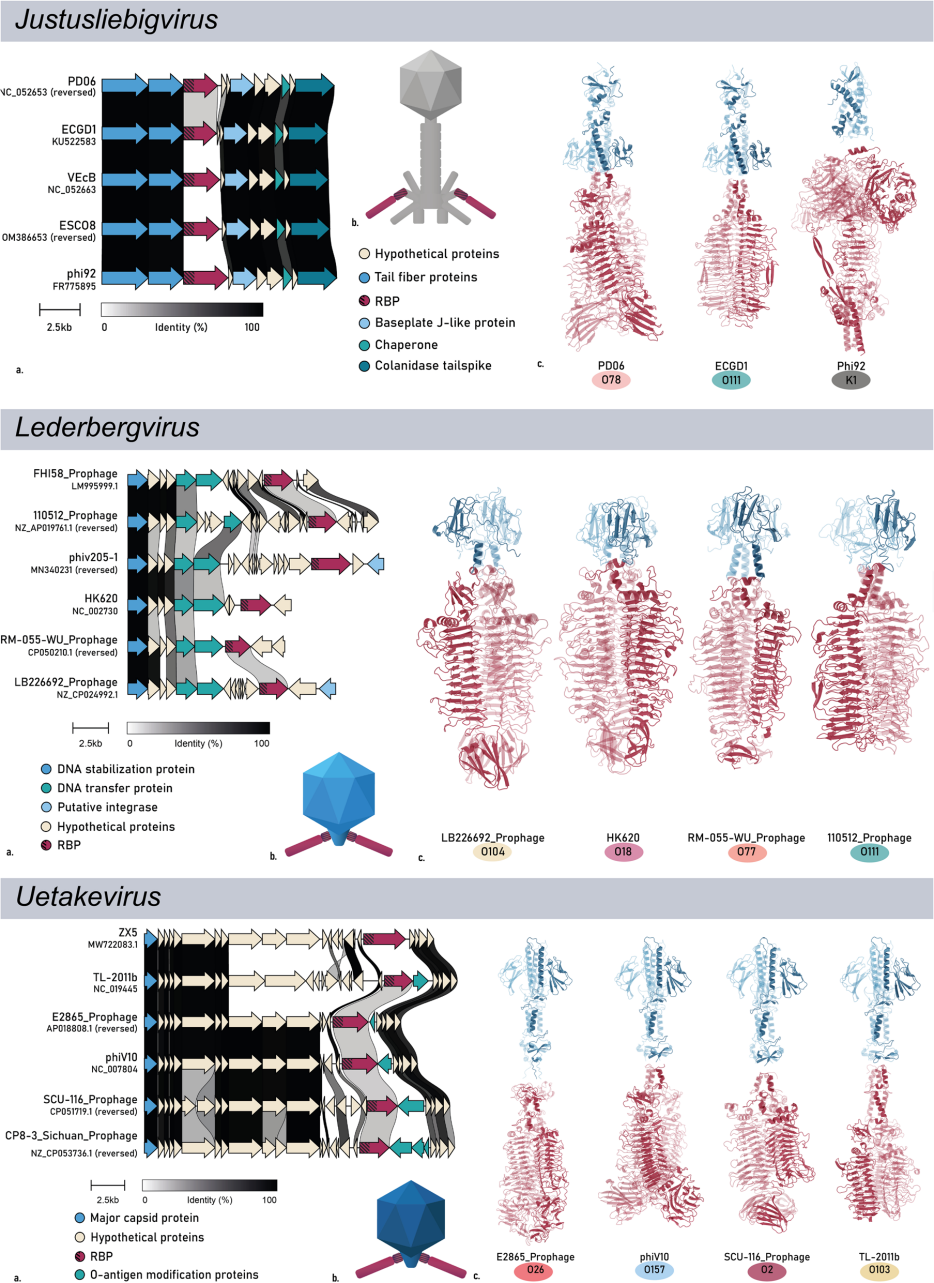

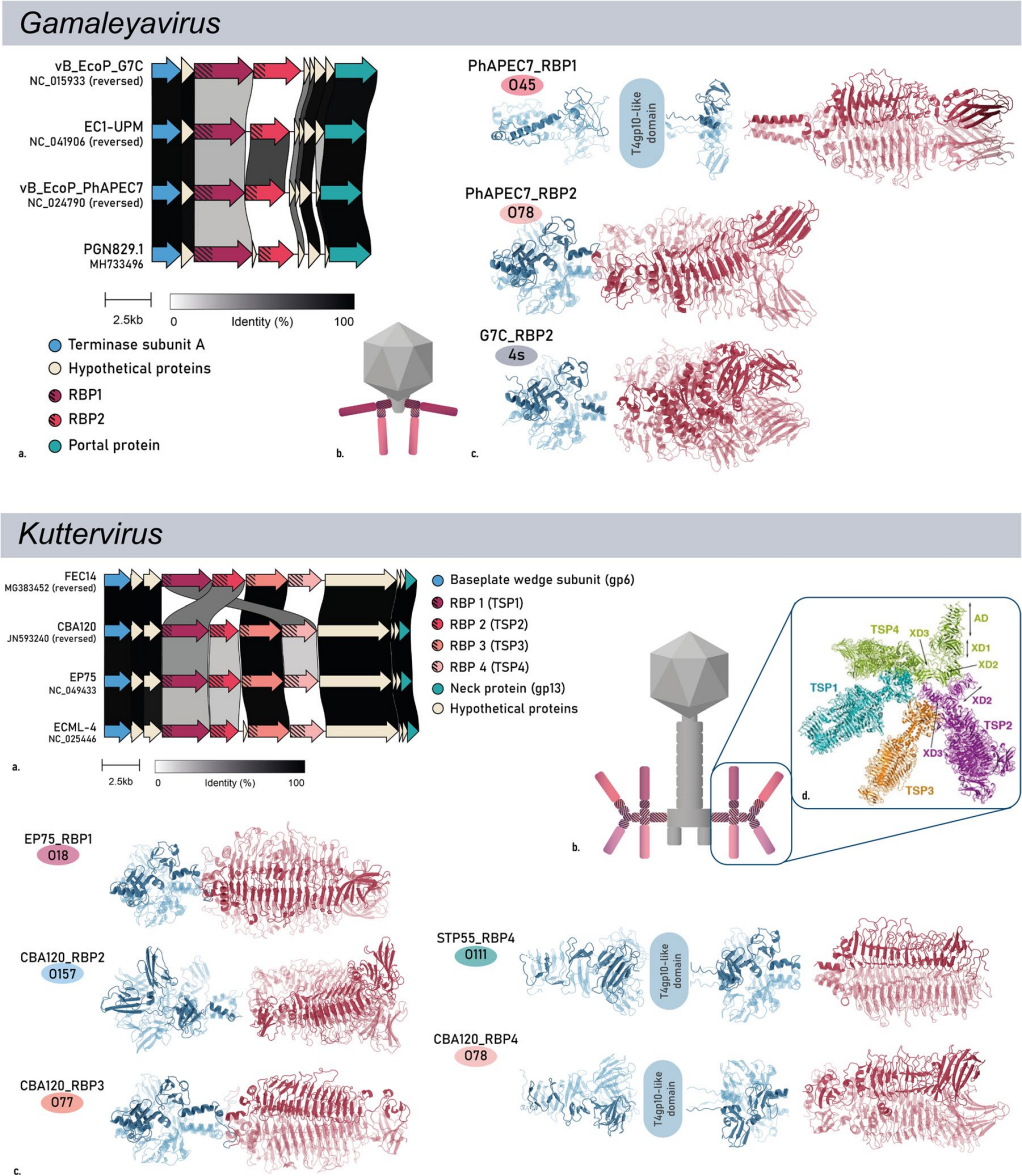
Overview on the RBP region and structure of selected RBPs from eight different phage genera (*Kayfuna-, Kaguna-, Nouzilly-, Justusliebig-, Lederberg-, Uetake-, Gamaleya-* and *Kutterviruses*). **a** Pairwise alignment of the RBP gene region of phage members with distinct RBDs within the genus. The different annotated genes are indicated. Within the RBP (red), the conserved anchor domains are highlighted with shading. **b** Simplified morphology of the RBP architecture of phages belonging to the respective genera with emphasis on the RBP and its two domains: anchor (shading) and RBD. **c** Predicted quaternary structure of selected genus members with the anchor domain and RBD highlighted in blue and red, respectively. **d** The branched structure of *Kuttervirus* RBPs, as illustrated by Sørensen et al. [56]. Sequence identity matrices for the separate domains can be found in Additional file 3: Figs. S1–S2

**Fig. 6:**
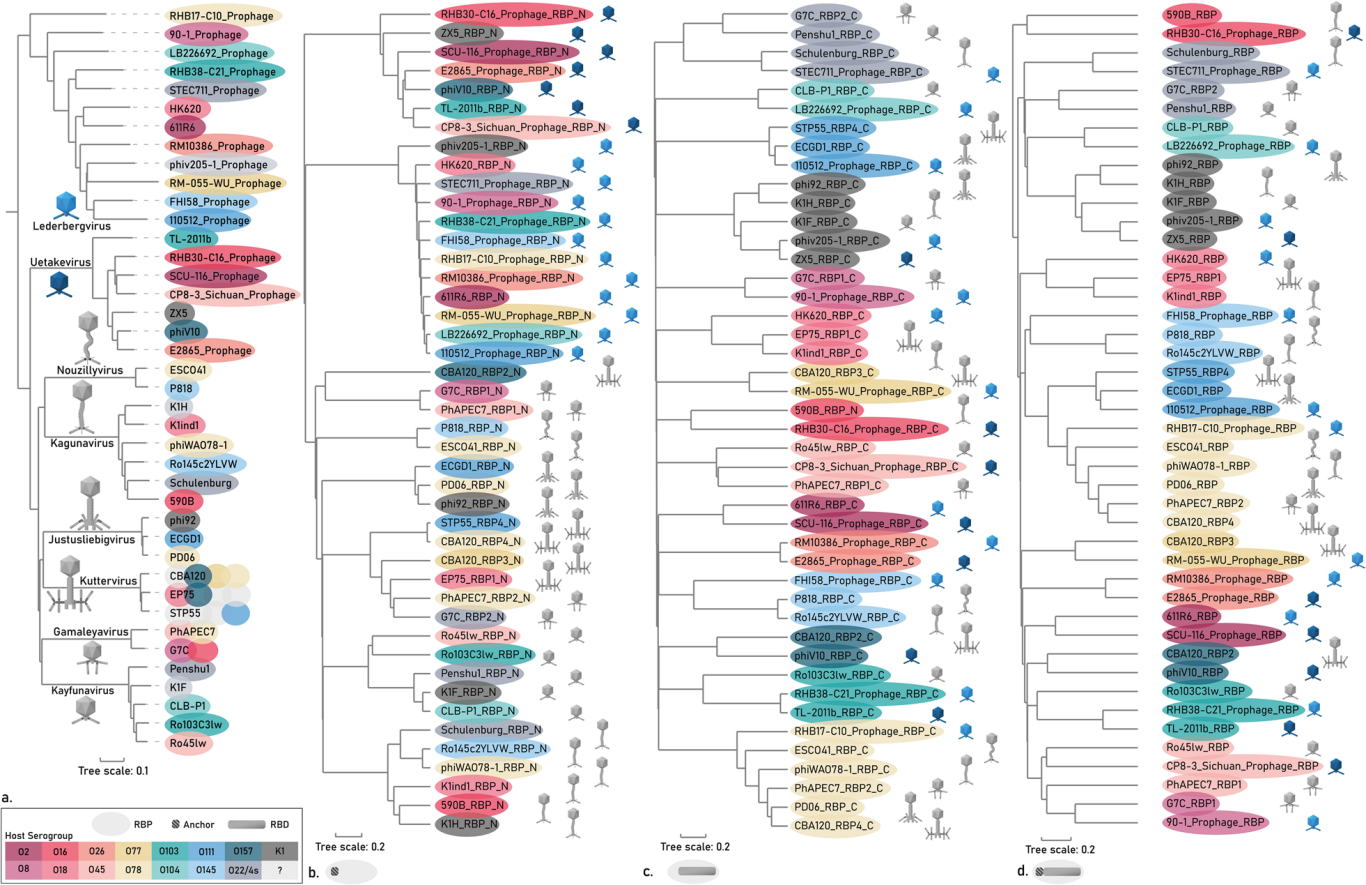
**a** Phylogenetic tree illustrating the taxonomic relationship of the various phages using VICTOR phylogeny on the whole phage genomes. The RBP subtype color scheme was illustrated according to their (predicted) host serogroup. When multiple RBPs were present in the phage genome, they are illustrated in the same order as the RBPs of their genera reference phages (CBA120 and G7C). Phylogenetic trees based on MAFFT G-INS-1 alignment of **b** the N-terminal anchor domains of the RBPs of interest, **c** the C-terminal RBD of the RBPs and **d** the complete RBP coding sequences to identify horizontal gene transfer events across taxonomic phage groups

In a final selection step we filtered for those genera that have at least two serogroup-specific RBPs, resulting in the omission of eight RBPs with an assigned O-antigen serogroup originating from eight different genera. After this validation and filtering, the final O-antigen specific RBP data set comprised 44 RBPs of 15 different RBP subtypes, distributed over phages belonging to eight phage genera with *Kayfunaviruses* (n = 4) and *Kutterviruses* (n = 4) having the most experimentally confirmed serogroup-specific RBPs (group A and B) whereas *Lederbergviruses* (n = 10) and *Uetakeviruses* (n = 6) count the most serogroup-predicted RBPs (Fig. 4b). The eight genera investigated in this research have distinct morphologies (Fig. 5b) and are not taxonomically related (Fig. 6a).

For each of the selected genera, phage genome synteny, RBP architecture, and structural analysis of the RBPs was performed (Fig. 5). The RBP coding sequences are generally the most variable sequences of the genome within a genus, embedded in a conserved synteny of genes, except for *Lederberg-* and *Uetakeviruses*. These genera show little to no similarities downstream of the RBP gene. This may be explained by the earlier observation that temperate phages are generally more subjected to horizontal gene transfer events [38]. All RBPs of the final data set have the classical N-terminal, conserved anchor domain with a C-terminal RBD, except for *Kayfunavirus* CLB_P1, where the RBP is split in two separate proteins, *i.e.*, an intermediate adapter protein (corresponding to the anchor) and a second protein (corresponding to the RBD) that is proposed to attach to the adapter protein (similar to phages K1-5, SP6, K1E [59] and KP34 [31]).

The branched RBP structures of *Gamaleya-* and *Kutterviruses* (Fig. 5b) appear to be highly receptive for HGT. From the sixteen selected *Gamaleyaviruses*, eleven distinct RBP subtypes were identified for RBP1 and seven for RBP2. Similarly, from the ten *E. coli* infecting *Kutterviruses*, five, one, four and five distinct RBP subtypes were found for RBP1, RBP2, RBP3 and RBP4, respectively. For *Gamaleyaviruses*, an *E. coli* serogroup was assigned to 28% of the RBP subtypes (Fig. 4b). The remaining RBP subtypes were not assigned to a serogroup either due to lack of data (28%) or due to serogroup inconsistencies among the RBP subtype members (50%). For *Kutterviruses*, 29.5% of RBP subtypes was assigned to an *E. coli* serogroup, whereas 41% were discarded due to a lack of data. The remaining RBP subtypes were discarded due to serogroup inconsistencies (29.5%), but, in contrary to other genera, mostly because the RBPs were predicted to target different species than *E. coli* (24%), among which many *Klebsiella pneumoniae* strains.

### Phylogenetic and structural analyses confirm the validity of the pipeline approach

Phylogenetic trees of the full phage genomes, the N-terminal anchors, the C-terminal RBDs and the full-length RBPs from the expanded data set are shown in Fig. 6. Again, a similar pattern is seen as for the initial data set (Fig. 3). The N-terminal anchors cluster according to taxonomy, whereas the C-terminal RBDs follow a serogroup clustering. The full-length RBPs also show a serogroup-driven clustering since C-terminal RBDs are the largest moiety of the RBP. Complementary to each phylogenetic tree based on MAFFT G-INS-1 alignment, amino acid similarities based on MUSCLE alignment of the RBPs and of the N-terminal and C-terminal domains of the final data set further confirm these findings (Additional file 3: Figures S1–S3). One exception to this perfect phylogenetic clustering, is the homology between N-terminal *Kutter-* and *Gamaleyavirus* domains. The domains of three out of four *Kuttervirus* RBPs (RBP1, RBP3 and RBP4) show high similarity to the domain of RBP2 of *Gamaleyaviruses*, and *Kuttervirus* RBP2 shows homology to *Gamaleyavirus* RBP1, indicating the conservation of the RBP branching structure across these genera [7, 21, 46].

**Fig. 7:**
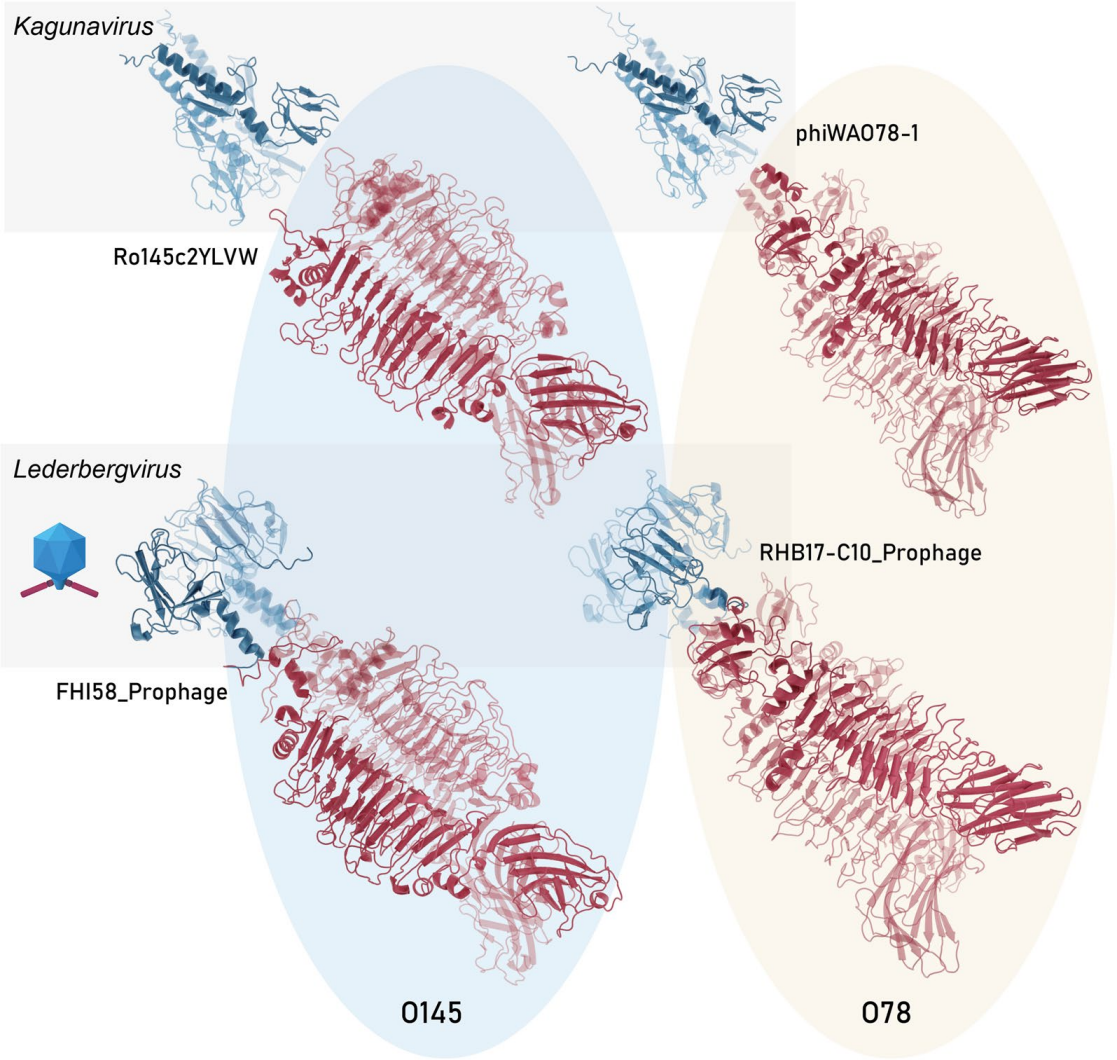
The quaternary structures of an exemplary subset of four RBPs from two genera (*Kaguna-* and *Lederbergvirus*) and two RBP subtypes (with assigned serogroups O145 and O78). The structure of the N-terminal anchor (protein structure in blue) is conserved at the phage genus level whereas the structure of the C-terminal RBD (protein structure in red) clusters per O-antigen serogroup, illustrating a HGT across genera

To further analyse the relationship between quaternary structure, serogroup specificity and phage genus, we predicted the quaternary structure of each RBP of the final data set with AlphaFold2, and clustered the structures per serogroup (Additional file 4: Fig. S4). Pairwise comparison of all structures also highlights HGT as the dominant principle, shaping evolution and structure of RBPs: the structure of the N-terminal anchor is conserved at the phage genus level, whereas the structure of the C-terminal RBD clusters per O-antigen serogroup, regardless of diverse primary sequences that can diverge up to 70%. As an illustration, this is clearly demonstrated for a systematic set of 2 × 2 RBPs belonging to *Kaguna-* and *Lederbergviruses*, targeting serogroups O78 and O145, respectively. Their anchor structures are highly similar at the genus level (RMSD of 1.46 and 1.18 Å), whereas the RBD structures are similar at the serogroup level (RMSD of 1.83 and 2.97 Å) (Fig. 7). This consistency in pattern revealed both by the phylogenetic clustering (Fig. 6) and the high conservation of the quartenary structure per function (Additional file 4: Fig. S4; Fig. 7) confirms the validity of the established pipeline to recruit and annotate newly predicted serogroup-specific RBPs and highlights again HGT as a major driver for specificity switches across taxonomic borders.

### Conserved DNA motifs in the RBP region potentially aid in swapping the RBD across lytic phage genera

**Fig. 8:**
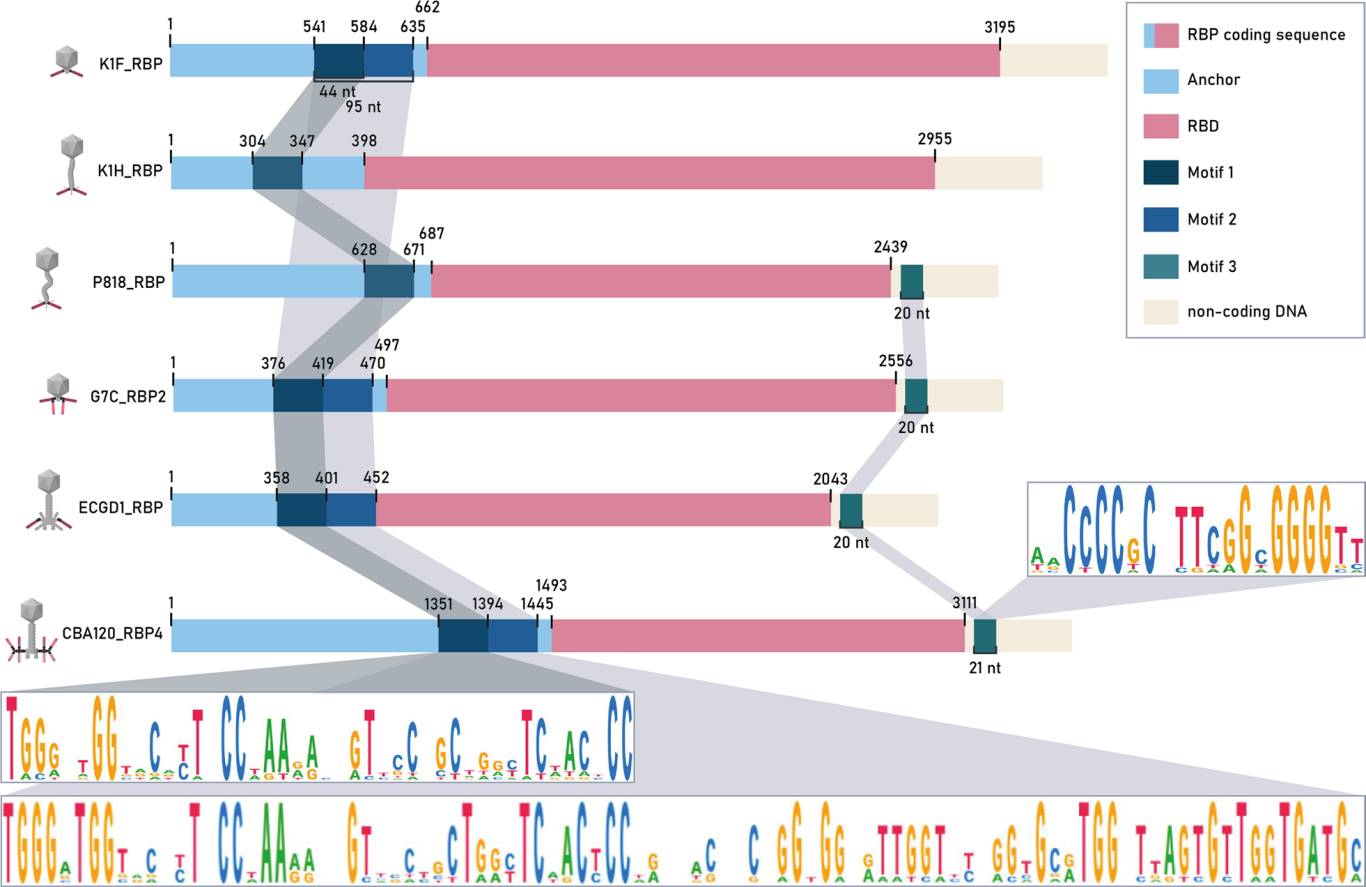
Visualization of the RBP sequence region of selected lytic phages belonging to the respective phage genera *Kayfuna-, Kaguna-, Nouzilly-, Gamaleya-, Justusliebig-* and *Kutterviruses*. One exemplary phage was chosen per genus to illustrate DNA sequence conservation across genera. Conserved motifs with lengths 44 (n = 20), 95 (n = 10) and 20 bp (n = 13) for respectively motif one to three show homology across the different phage genera. The consensus sequences are shown through sequence logos

Through further investigation, two conserved DNA sequence regions were identified, located before and after the RBD. Remarkably, these conserved regions are present across RBP genes from all lytic phage genera *Gamaleya-, Kaguna-, Kayfuna-, Kutter-, Justusliebig-* and *Nouzillyviruses* (Fig. 8) but not in the prophages belonging to the *Uetake-* and *Lederbergviruses*. Motif one comprises a 44 nt long region located at the end of the anchor domain sequence and is conserved across all six *Kagunavirus* RBPs, four out of five *Kayfunavirus* RBPs, all three *Justusliebigvirus* RBPs, both *Nouzillyvirus* RBPs, the second RBP of both *Gamaleyaviruses* and all *Kuttervirus* RBPs except for CBA120 RBP2 (Additional file 5: Fig. S5, a; Additional file 6: Fig. S6) (n = 20). In a subset of these RBPs (n = 10) this motif is conserved over a longer stretch (motif two; 95 nt) (Additional file 5: Fig. S5, b; Additional file 6: Fig. S7). Motif three on the other hand, is located in the noncoding region downstream of the RBP coding sequence and is conserved for all *Gamaleya-, Kutter-* and *Justusliebigvirus* RBPs, one *Kayfunavirus* and one *Nouzillyvirus* RBP (20 nt; n = 13; Additional file 5: Fig. S5c; Additional file 6: Fig. S8). This conserved motif is also predicted to function as a terminator sequence. Average DNA sequence identities of 63 ± 9, 69 ± 8 and 81 ± 12% were obtained for the respective motifs. Conservation within these motifs is significantly higher than in the surrounding DNA sequence regions (Additional file 5: Fig. S5d). While recombination can take place in a non-homology and homology manner, we suggest that these conserved regions up- and downstream of the RBD coding sequences across taxonomic boundaries may act as recombination hotspots to quickly drive niche speciation by acquiring a suitable RBD from another phage through horizontal transfer, even across taxonomic borders.

## Discussion

We built a pipeline to identify serogroup-specific RBPs in silico. We therefore relied on the modularity principle of RBPs that retain conserved anchors for structural attachment to the phage tail, while swapping the RBD for specificity switches. Both at the phylogenetic and the structural level this modularity gives a clear guidance in classifying the RBPs in RBP subtypes. In total, 14 different RBP subtypes targeting O2, O8, O16, O18, 4s/O22, O26, O45, O77, O78, O103, O104, O111, O145 and O157 were identified in 39 phages spread over eight different phage genera. Simultaneously, several clustered RBP subtypes were found that most likely target a different receptor than the O-antigen. For example, during the serogroup validation step, the RBP of *Justusliebigvirus* VecB showed similarity to RBPs from prophages integrated in *E. coli* strains of serogroups O6, O11 and O153 with 89.8, 66.1 and 61.7% aa identity. Also, RBP1 of *Gamaleyavirus* PGN829.1 shows more than 99.5% aa identity with RBPs from prophages in strains with serogroups O11, O83, O86 and O102. Other examples are the RBP of *Kayfunavirus* YZ1 (serogroups O102, O6, O1, O153 and O6; ≥ 95% identity), the RBP of *Uetakevirus* phiv142-3 (including serogroups O5, O1, O102 and O51; ≥ 95% identity), and the RBP of *Justusliebigvirus* alia (including serogroups O7, O23, O146; ≥ 75.7% identity). A RBP binding smooth *E. coli* strains from multiple serogroups was identified previously [20]. One possible explanation is that these RBPs belonging to the same subtype target a receptor that is shared across multiple serogroups, such as the K-antigen (capsule). Outer membrane proteins may be less likely to serve as receptor since RBPs of phages infecting smooth strains cannot easily approach the outer membrane proteins due to steric hindrance of the long chain O-antigen [8, 20, 29]. Secondly, these RBPs belonging to the same RBP subtype could potentially have further diverged to alter their host specificity through single point mutations in their substrate binding site, as has been described for some tail fibers [3, 33, 60, 66]. Further investigation is needed to draw any further conclusion, but multiple serogroup-targeting phages may have a broader therapeutic potential, which is an attractive trait for the development of phage cocktails.

The temperate phage genera *Lederberg-* and *Uetakeviruses* offer an elegant avenue to identify new RBPs with specificity towards an O-antigen serogroup of interest. Phages of these genera were identified in eight out of nine serogroups of interest and there is a clear link between the RBP of the prophage and the O-antigen serogroup of their host [5]. This approach is generic and can be easily expanded to other serogroups. In addition to our findings, the RBP sequences of *Salmonella enterica* infecting *Lederbergviruses* have been used to predict the O-antigen type of its host, with 743 prophage RBPs clustering into 18 distinct RBP subtypes correlating perfectly with the O-antigen polysaccharide that its host displays on its surface [5]. However, one limitation of this approach is that some *Lederberg-* and *Uetakeviruses* may also encode an O-antigen modification gene behind their RBP [9, 44], thereby changing the receptor as a mechanism to prevent superinfection. Next to serogroup prediction, RBDs of *Lederberg-* and *Uetakeviruses* with a podovirus morphotype have been successfully grafted into myo-like phage tail-like bacteriocins (PTLBs) [49, 50] to successfully swap the killing spectrum of the PTLB. In addition, many RBDs of RBPs of *Kutterviruses* share homology to those of *Lederberg-* or *Uetakeviruses*, such as TSP3 of phage SPTD1 [18] and to other RBDs identified in this work. This shows that *Lederberg-* and *Uetakeviruses* are ideal candidates as a start point to identify a RBP targeting an O-antigen serogroup of interest and expand from there to recruit more RBPs belonging to the same RBP subtype from phages belonging to other taxonomic groups.

Our research suggests that many phages belonging to the genera *Gamaleya-, Justusliebig-, Kaguna-, Kayfuna-, Kutter-, Lederberg-, Nouzilly-* and *Uetakeviruses* have their RBP(s) as the sole factor determining serogroup specificity. Consequently, these RBPs can be used to predict the phage host serogroup relying on the conservation of serogroup specificity of RBP subtypes. *Kutterviruses* have previously been used to predict the host serogroup of *Salmonella enterica* and *E. coli.* RBP subtypes (> 75% aa identity) were confirmed for the O78 antigen of *E. coli* and the O22 antigen and O4/O9 antigen backbone of *S. enterica* [56]. In our work, we found reliable clustering in RBP subtypes based on mere ≥ 30% aa identity, while the predicted quaternary protein structures remain highly similar. This indicates that substantial divergence by adaptive evolution happens to improve phage fitness upon a HGT event of a RBD, while conserving serogroup specificity. RBPs of the same subtype but with low sequence similarity thus have a more distantly related ancestor compared to RBPs with higher similarities. These observations illustrate the interplay of horizontal and vertical evolutionary processes that shape tailspikes. However, the low threshold may lead to the inclusion of false positives, when assigning a serogroup to a RBP that has already undergone crucial mutations resulting in a serogroup specificity switch. As a criterion, we stated that 90% of all RBPs within a subtype needed to be conform in their host serogroup, otherwise the RBP subtype was classified as non-O-antigen targeting. Therefore, we may have falsely discarded serogroup-specific RBP subtypes due to a single RBP that has potentially alternated its specificity. In addition to the eight genera investigated in this study, *Agtre-, Phapecocta-, Roguna-* and *Vectreviruses* and members of the family *Ackermannviridae* or subfamily *Braunvirinae* also frequently popped up in the group F RBPs based on HGT identification, suggesting that they could also play an important role in the HGT of RBPs with *E. coli* serogroup specificity.

Members of these genera may be engineered to swap the host range of the phages simply by exchanging the RBD domains. As phages seem to have switched host range on many occasions throughout evolution by horizontal transfer, phages could be designed with adapted RBPs to target the strain of choice. *Przondovirus* K11, a phage related to *Kayfunaviruses*, has been successfully engineered by swapping the RBD to alter the host range towards different *Klebsiella* capsular serotypes [32]. Similarly, *Kuttervirus* phage SPTD1 RBDs have been swapped within the same phage genus to target different *Salmonella* O-antigen serogroups [18]. Additionally, the RBDs of podo-like *Lederberg-* and *Uetakeviruses* have been exchanged with myo-like PTLBs as mentioned previously, illustrating that RBDs can be exchanged across different morphologies [49, 50].

The observed sequence conservation surrounding the RBD may aid recombination across *Gamaleya-, Justusliebig-, Kaguna-, Kayfuna-, Kutter-* and *Nouzillyviruses*. Although illegitimate recombination events can happen virtually anywhere in the phage genome, certain regions of sequence conservation can serve as recombination hotspots. Such hotspots have been identified on multiple occasions. In temperate phage clusters including *Lederbergviruses*, conserved sequence motifs were identified between genome cassettes, resulting in higher genome mosaicism [6, 9, 25, 47]. Moreover, sequence homology has also been identified across different genera of lytic phages. For example, sequence homology between the different RBPs of *Kutterviruses* and between *Kutter-* and *Gamaleyaviruses* have already been suggested to aid recombination across different tailspike genes [7, 21, 46, 56]. In this research we observed conserved motifs that may allow homologous recombinations to occur at a higher rate in the sequence regions surrounding the RBD in up to six different lytic phage genera. Additionally, when expanding the data set in this project, various HGT events were observed across phages belonging to the same, recurring genera, indicating higher odds for HGT events within the RBPs across these genera than to other genera. However, these motifs are not universal for all lytic phage RBPs in the final data set and no correlation could be observed between the presence of these motifs and the number of recombination events that we observed between these phages.

A few hurdles were identified when performing this research. (i) The first limitation is the lack of phage–host serogroup data in public databases. When the serogroup of the phage host is known, it should be mentioned as it can offer valuable information in phage–host interaction studies. Additionally, the number of available phage genomes of phages infecting smooth *E. coli* strains is relatively low compared to those infecting rough *E. coli* strains. On top of that, most of the smooth *E. coli* infecting phage genomes that are available infect *E. coli* serogroup O157. To find new phages, smooth *E. coli* strains of all serogroups should be used more frequently as hosts during phage isolation. The method used for *E. coli* serotyping is also relevant information, since additional O-antigen modification genes can be encoded by prophages, which can be missed by genetic-based serotyping assays. (ii) A second hurdle is the incorrect annotation of many RBPs in databases such as NCBI. This is partially due to the variety of used terminology. Tail fibers generally have a fiber-like structure dominated by a long α-helix bundle with a C-terminal RBD, whereas tailspikes have an enzymatically active, β-helical, elongated structure with no, one or two C-terminal carbohydrate-binding or chaperone domains [12]. Both terms are often mixed. Wrongly annotated RBPs cause the need for manual and time-consuming curation of the RBP through phage genome alignments. New computational tools such as PhageDPO [63] may facilitate this process, but still require manual validation. (iii) The number of RBP structures defined by crystallography is growing but still scarce. Therefore, we extensively relied on the AlphaFold2 algorithm to reveal the remarkably conserved anchor and RBD quaternary structures, corresponding to genus and serogroup, respectively. Yet, the AlphaFold2 algorithm frequently failed in delivering good structures, such as the trimeric structure of O8 and O16 targeting RBPs, either due to limitations in computing power to deal with these large, trimeric proteins or due to high error estimates. The limitation in computing power could mostly be circumvented by using high computing infrastructure and splitting the RBP in its anchor and RBD for separate predictions. The high error estimates are caused by the incapacity to predict the mutual orientation of the separate domains because of the flexible hinge domains, due to the limited number of available crystal structures (e.g., for the anchor domain of the *Lederbergvirus* RBPs), but also due to the intervening T4gp10-like domains that are needed to create branched RBPs [31, 45, 46]).

In sum, a pipeline to identify and validate *E. coli* O-antigen specific RBPs was established. Eight phage genera (*Gamaleya-, Justusliebig-, Kaguna-, Kayfuna-, Kutter-, Lederberg-, Nouzilly-* and *Uetakeviruses*) emerged for their high proportion of serogroup-specific RBPs. With their conserved N-terminal anchor domain and exchangeable RBD, they offer an ideal platform for phage host engineering in terms of O-antigen serogroup specificity. This research also emphasizes the need to study recombination hotspots surrounding RBDs that might lead to a better understanding of phage genome mosaicism.

### Supplementary Information

**Additional file 1**. **Table S1**: Overview of the phages used in this study.

**Additional file 2**. **Table S2**: *E*. *coli* strains encoding temperate phages belonging to genera Uetakevirus or Lederbergvirus.

**Additional file 3**. **Figures S1**–**S3**: Similarity matrices of the RBP and its domains after MUSCLE multiple sequence alignment.

**Additional file 4**. **Figure S4**: Predicted quaternary structures of the phage RBPs using AlphaFold2.

**Additional file 5**. **Figure S5**: DNA sequence motifs surrounding the RBD show higher sequence conservation than surrounding regions.

**Additional file 6**. **Figures S6**–**S8**: Multiple sequence alignments of the motif DNA sequences of the RBPs of phages in the final data set.

## Data Availability

The datasets supporting the conclusions of this article are included within the article and its additional files.
