# Supplementary material for: Phage tailspike modularity and horizontal gene transfer reveals specificity towards E. coli O-antigen serogroups
Source: Virol J. 2023 Aug 7;20:174. doi: 10.1186/s12985-023-02138-4 (PMC10408124; doi:10.1186/s12985-023-02138-4)
Supplement: Supplementary file 1 — Additional file 1. Table S1: Overview of the phages used in this study. [file 12985_2023_2138_MOESM1_ESM.docx]

# Additional file 1

**Table S1: Overview of the phages used in this study.**

| **Dataset** | **Phage  genus/subfamily** | **RBP name** | **Phage/strain  accession** | **Tailspike  accession** | **Receptor  (serogroup)** | **Serogroup  specificity** | **Ref** | **Delineation  (aa)** |
| --- | --- | --- | --- | --- | --- | --- | --- | --- |
| Group A | Gamaleyavirus | G7C_RBP2 | NC_015933.1 | YP_004782195.1 | 4s/O22 | RBP | (1) | 166 |
| Group A | Kagunavirus | K1H_RBP | NC_027994.1 | YP_009168860.1 | K1 | RBP | (2) | 133 |
| Group A | Kayfunavirus | K1F_RBP | NC_007636.1 | YP_424959.1 | K1 | RBP | (3) | 221 |
| Group A | Kuttervirus | CBA120_RBP2 | JN593240 | AEM91897.1 | O157 | RBP | (4) | 259 |
| Group A | Kuttervirus | CBA120_RBP3 | JN593240 | AEM91898.1 | O77 | RBP | (4) | 184 |
| Group A | Kuttervirus | CBA120_RBP4 | JN593240 | AEM91899.1 | O78 | RBP | (4) | 498 |
| Group A | Kuttervirus | EP75_RBP1 | NC_049433.1 | YP_009880322.1 | O18 | RBP | (5) | 181 |
| Group A | Lederbergvirus | HK620_RBP | NC_002730.1 | NC_002730.1 | O18 | RBP | (6) | 124 |
| Group A | Lederbergvirus | LB226692_Prophage_RBP | GCA_000215685.3 | / | O104 | RBP | (7) | 123 |
| Group A | Uetakevirus | phiV10_RBP | NC_007804.2 | YP_512279.1 | O157 | RBP | (8) | 205 |
| Group B | Kagunavirus | Ro145c2YLVW_RBP | MH051334 | AVZ45589.1 | O145 | Phage | (9) | 124 |
| Group B | Kayfunavirus | CLB_P1_RBP | KC109329.1 | AGD81076.1, AGD81077.1 | O104 | Phage | (10) | / |
| Group B | Kayfunavirus | Ro103C3Iw_RBP | MN067430.1 | QDH94159.1 | O103 | Phage | (11) | 227 |
| Group B | Kayfunavirus | Ro45lw_RBP | NC_048136.1 | YP_009818296.1 | O45 | Phage | (12) | 171 |
| Group C | Gamaleyavirus | PhAPEC7_RBP2 | NC_024790.1 | YP_009056200.1 | O78 | HGT | (13) | 235 |
| Group C | Kagunavirus | phiWAO78-1_RBP | MW331437.1 | QQV88057.1 | O78 | HGT | (14) | 124 |
| Group C | Kayfunavirus | Penshu1_RBP | MK903281.1 | QEG09817.1 | 4s/O22 | HGT | (15) | 221 |
| Group C | Lederbergvirus | phiv205-1_RBP | MN340231.1 | QGF19902.1 | K1 | HGT | (16) | 59 |
| Group C | Nouzillyvirus | ESCO41_RBP | NC_047820.1 | YP_009789960.1 | O78 | HGT | (14) | 236 |
| Group C | Uetakevirus | TL-2011b_RBP | NC_019445 | YP_007001999.1 | O103 | HGT | (17) | 205 |
| Group D | Lederbergvirus | 110512_Prophage_RBP | NZ_AP019761.1 | / | O111 | HGT |  | 123 |
| Group D | Lederbergvirus | FHI58_Prophage_RBP | LM995999.1 | / | O145 | HGT |  | 126 |
| Group D | Lederbergvirus | RHB38-C21_Prophage_RBP | CP057085.1 | / | O103 | HGT |  | 123 |
| Group D | Lederbergvirus | RM10386_Prophage_RBP | NZ_CP028126.1 | / | O26 | HGT |  | 126 |
| Group D | Uetakevirus | CP8-3_Sichuan_Prophage_RBP | CP053736 | / | O45 | HGT |  | 206 |
| Group D | Uetakevirus | E2865_Prophage_RBP | AP018808.1 | / | O26 | HGT |  | 206 |
| Group D | Uetakevirus | MINF_2E-sc-2280463_Prophage_RBP | LR890651.1 | / | X9 | No |  |  |
| Group D | Uetakevirus | RHB38-C01_Prophage_RBP | CP057104.1 | / | X11 | No |  |  |
| Group D | Uetakevirus | RHBSTW-00777_Prophage_RBP | CP056165.1 | / | X10 | No |  |  |
| Group E | Caminolopintovirus | HC6_RBP | OL362274.1 | URY99407.1 | X12 | No |  |  |
| Group E | Gamaleyavirus | EC1-UPM_RBP1 | NC_041906.1 | YP_009598345.1 | X30 | No |  |  |
| Group E | Gamaleyavirus | G7C_RBP1 | NC_015933.1 | YP_004782196.1 | O8 | HGT |  | 374 |
| Group E | Gamaleyavirus | IME11_RBP1 | NC_019423.1 | YP_006990695.1 | X31 | No |  |  |
| Group E | Gamaleyavirus | PD205_RBP1 | ON922919.1 | UVK80510.1 | X36 | No |  |  |
| Group E | Gamaleyavirus | PD205_RBP2 | ON922919.1 | UVK80511.1 | X25 | No |  |  |
| Group E | Gamaleyavirus | PD38_RBP1 | MH669274.1 | AXY81356.1 | X18 | No |  |  |
| Group E | Gamaleyavirus | PGN829.1_RBP1 | MH733496.1 | AXY82583.1 | X3 | No |  |  |
| Group E | Gamaleyavirus | PGN829.1_RBP2 | MH733496.1 | AXY82585.1 | X2 | No |  |  |
| Group E | Gamaleyavirus | PhAPEC5_RBP1 | NC_024786.1 | YP_009055578.1 | X33 | No |  |  |
| Group E | Gamaleyavirus | PhAPEC7_RBP1 | NC_024790.1 | YP_009056201.1 | O45 | HGT |  | 376 |
| Group E | Gamaleyavirus | SP5M_RBP1 | MT682708.1 | QLF80668.1 | X34 | No |  |  |
| Group E | Gamaleyavirus | SP5M_RBP2 | MT682708.1 | QLF80674.1 | X26 | No |  |  |
| Group E | Gamaleyavirus | St11Ph5_RBP1 | MG208881.1 | ATS92541.1 | X1 | No |  |  |
| Group E | Gamaleyavirus | St11Ph5_RBP2 | MG208881.1 | ATS92539.1 | X32 | No |  |  |
| Group E | Gamaleyavirus | ZQ2_RBP1 | MW630115.1 | QWY13162.1 | X35 | No |  |  |
| Group E | Gamaleyavirus | ZQ2_RBP2 | MW630115.1 | QWY13163.1 | X29 | No |  |  |
| Group E | Justusliebigvirus | PHB05_RBP | NC_052652.1 | YP_009984503.1 | X45 | No |  |  |
| Group E | Kagunavirus | 26_RBP | MZ832314.1 | UAW06957.1 | X11 | No |  |  |
| Group E | Kagunavirus | 590B_RBP | MW722821.1 | QVR48605.1 | O16 | HGT |  | 132 |
| Group E | Kagunavirus | fBC-Eco01_RBP | OM272052.1 | UMO77109.1 | X1 | No |  |  |
| Group E | Kagunavirus | fFiEco02_RBP | MT711523.1 | QNO11594.1 | X16 | No |  |  |
| Group E | Kagunavirus | Golestan_RBP | NC_042084.1 | YP_009620110.1 | X18 | No |  |  |
| Group E | Kagunavirus | HSE2_RBP | MG252615.1 | AUE23473.1 | X17 | No |  |  |
| Group E | Kagunavirus | K1ind1_RBP | NC_041897.1 | YP_009597310.1 | O18 | HGT | (2) | 125 |
| Group E | Kagunavirus | Schulenburg_RBP | MK931438.1 | QEG06822.1 | 4s/O22 | HGT |  | 124 |
| Group E | Kagunavirus | ST20_RBP | MF153391.1 | ASH99372.1 | X15 | No |  |  |
| Group E | Kagunavirus | ZCEC5_RBP | MK542015 | QBJ02991.1 | X19 | No |  |  |
| Group E | Kayfunavirus | CY1_RBP | OM937123.1 | UOL49326.1 | X22 | No |  |  |
| Group E | Kayfunavirus | ECG4_RBP | MN218775.1 | QFG07004.1 | X23 | No |  |  |
| Group E | Kayfunavirus | EcoDS1_RBP | NC_011042.1 | YP_002003781.1 | X24 | No |  |  |
| Group E | Kayfunavirus | IMEP24_RBP | MZ648215.1 | UCR92084.1 | X18 | No |  |  |
| Group E | Kayfunavirus | IMM-002_RBP | NC_048071.1 | YP_009812899.1 | X7 | No |  |  |
| Group E | Kayfunavirus | LM33_P1_RBP | NC_031937.1 | YP_009324518.1 | X18 | No |  |  |
| Group E | Kayfunavirus | LS2_RBP | MN518894.1 | QLF86424.1 | X12 | No |  |  |
| Group E | Kayfunavirus | P762_RBP | MW876471.1 | QUR34677.1 | X13 | No |  |  |
| Group E | Kayfunavirus | PE3-1_RBP | NC_024379.1 | YP_009044291.1 | X20 | No |  |  |
| Group E | Kayfunavirus | Peacock_RBP | MK903279.1 | QEG09711.1 | X14 | No |  |  |
| Group E | Kayfunavirus | PHB19_RBP | MN481365.1 | QHI00738.1 | X21 | No |  |  |
| Group E | Kayfunavirus | SP7_RBP | MT682707.1 | QLF80618.1 | X26 | No |  |  |
| Group E | Kayfunavirus | ST31_RBP | NC_047829.1 | YP_009790654.1 | X25 | No |  |  |
| Group E | Kayfunavirus | Vec13_RBP | MH400309.1 | AXF38930.1 | X27 | No |  |  |
| Group E | Kayfunavirus | YZ1_RBP | NC_047927.1 | YP_009798557.1 | X28 | No |  |  |
| Group E | Kuttervirus | 3HA11_RBP4 | MN342150.1 | QFR58461.1 | X42 | No |  |  |
| Group E | Kuttervirus | 4HA11_RBP4 | MN445184.1 | QKE54815.1 | X43 | No |  |  |
| Group E | Kuttervirus | CBA120_RBP1 | JN593240 | AEM91896.1 | X41 | No |  |  |
| Group E | Kuttervirus | ECML-4_RBP1 | NC_025446 | YP_009101515.1 | X40 | No |  |  |
| Group E | Kuttervirus | ECML-4_RBP3 | NC_025446 | YP_009101512.1 | X2 | No |  |  |
| Group E | Kuttervirus | EP75_RBP3 | NC_049433.1 | YP_009880320.1 | X37 | No |  |  |
| Group E | Kuttervirus | EP75_RBP4 | NC_049433.1 | YP_009880319.1 | X38 | No |  |  |
| Group E | Kuttervirus | FEC14_RBP4 | MG383452 | ATW66762.1 | X39 | No |  |  |
| Group E | Kuttervirus | PhaxI_RBP1 | NC_019452 | YP_007002807.1 | X44 | No |  |  |
| Group E | Kuttervirus | Sa157lw_RBP1 | MH427377.1 | AXF39258.1 | X46 | No |  |  |
| Group E | Kuttervirus | Sa157lw_RBP3 | MH427377.1 | AYC62343.1 | X47 | No |  |  |
| Group E | Lederbergvirus | 611R6_RBP | ON470589.1 | URC09205.1 | O2 | HGT |  | 124 |
| Group E | Lederbergvirus | APC_JM3.2_RBP | MG197996.1 | ATN92736.1 | X4 | No |  |  |
| Group E | Lederbergvirus | E212_RBP | MZ043897.1 | QVW53997.1 | X5 | No |  |  |
| Group E | Lederbergvirus | HF4s_RBP | MT833387.1 | QOI58119.1 | X14 | No |  |  |
| Group E | Lederbergvirus | Gally_RBP | OV696608.1 | CAH1189517.1 | X3 | No |  |  |
| Group E | Lederbergvirus | Kapi1_RBP | MT813197.1 | QOC59583.1 | X6 | No |  |  |
| Group E | Nouzillyvirus | CJ19_RBP | MT176427.1 | QIW88869.1 | X48 | No |  |  |
| Group E | Nouzillyvirus | P817_RBP | MZ826699.1 | UAV84693.1 | X49 | No |  |  |
| Group E | Nouzillyvirus | P818_RBP | ON062054.1 | UOX38473.1 | O145 | HGT |  | 229 |
| Group E | Uetakevirus | phiv142-3_RBP | MN187550.1 | QGF19612.1 | X1 | No |  |  |
| Group E | Uetakevirus | ZX5_RBP | MW722083.1 | QTH80017.1 | K1 | HGT |  | 174 |
| Group F | Aglimvirinae | fGh-Ecl02_RBP1 | ON212266.1 | USL85812.1 | O8 | HGT |  |  |
| Group F | Agtrevirus | MK-13_RBP1 | NC_049455.1 | YP_009882837.1 | O8 | HGT |  |  |
| Group F | Agtrevirus | MK-13_RBP2 | NC_049455.1 | YP_009882838.1 | X21 | No |  |  |
| Group F | Agtrevirus | P46FS4_RBP1 | NC_049509.1 | YP_009889344.1 | X14 | No |  |  |
| Group F | Autographiviridae | Yanou_RBP | ON568193.1 | UTQ78082.1 | X21 | No |  |  |
| Group F | Braunvirinae | JeanPiccard_RBP | MZ501080.1 | QXV80800.1 | O2 | HGT |  |  |
| Group F | Braunvirinae | RTP_RBP | AM156909.1 | CAJ42254.1 | X21 | No |  |  |
| Group F | Buchananvirus | Sa179lw_RBP | NC_054637.1 | YP_010053157.1 | X15 | No |  |  |
| Group F | Chaseviridae | BUCT696_RBP | OL770365.1 | UKH48828.1 | X36 | No |  |  |
| Group F | Deseoctovirus | S192_RBP | ON239132.1 | URP83754.1 | X19 | No |  |  |
| Group F | Guernseyvirinae | VLCpiS11a_RBP | NC_069154.1 | YP_010582687.1 | X26 | No |  |  |
| Group F | Jillinvirus | ep3_RBP | KM360178.1 | AIM50567.1 | O78 | HGT |  |  |
| Group F | Justusliebigvirus | ECGD1_RBP | KU522583.1 | AMM43477.1 | O111 | HGT |  | 151 |
| Group F | Justusliebigvirus | ESCO8_RBP | OM386653.1 | UPW37474.1 | X8 | No |  |  |
| Group F | Justusliebigvirus | PD06_RBP | NC_052653.1 | YP_009984702.1 | O78 | HGT |  | 151 |
| Group F | Justusliebigvirus | phi92_RBP | FR775895.2 | CBY99572.1 | K1 | RBP | (18) | 80 |
| Group F | Justusliebigvirus | VEcB_RBP | NC_052663.1 | YP_009987349.1 | X7 | No |  |  |
| Group F | Kayfunavirus | ZG49_RBP | NC_047777.1 | YP_009787294.1 | X16 | No |  |  |
| Group F | Koutsourovirus | phiKDA1_RBP | NC_027980.1 | YP_009167707.1 | O77 | HGT |  |  |
| Group F | Kuravirus | ES17_RBP | MN508615.2 | QPL11059.1 | X18 | No |  |  |
| Group F | Kuttervirus | LPST94_RBP1 | MH523359.1 | AXF41694.1 | X25 | No |  |  |
| Group F | Kuttervirus | STP55_RBP4 | OM688977.1 | UPU15645.1 | O111 | HGT |  | 497 |
| Group F | Lederbergvirus | 90-1_Prophage_RBP | CP050047.1 | / | O8 | HGT |  | 128 |
| Group F | Lederbergvirus | FORC_031_Prophage_RBP | NZ_CP013190.1 | / | X7 | No |  |  |
| Group F | Lederbergvirus | K71-77_Prophage_RBP | CP040886.1 | / | X8 | No |  |  |
| Group F | Lederbergvirus | RHB17-C10_Prophage_RBP | CP057690.1 | / | O78 | HGT |  | 123 |
| Group F | Lederbergvirus | RM-055-WU_Prophage_RBP | CP050210.1 | / | O77 | HGT |  | 125 |
| Group F | Lederbergvirus | STEC711_Prophage_RBP | CP041416.1 | / | 4s/O22 | HGT |  | 123 |
| Group F | Matsuvirus | pYD6-A_RBP | NC_020849.1 | YP_007674303.1 | X18 | No |  |  |
| Group F | Phapecoctavirus | Phapec8_RBP | NC_020079.1 | YP_007348539.1 | K1 | HGT |  |  |
| Group F | Phapecoctavirus | SKA64_RBP | OM362897.1 | UKM17348.1 | 4s/O22 | HGT |  |  |
| Group F | Phapecoctavirus | Ro121c4YLVW_RBP | NC_052654.1 | YP_009984889.1 | X25 | No |  |  |
| Group F | Rogunavirus | Jk06_RBP | NC_007291.1 | YP_277515.1 | O157 | Phage | (19) |  |
| Group F | Shuimuvirus | IME207_RBP | NC_031924.1 | YP_009322784.1 | K1 | HGT |  |  |
| Group F | Studiervirinae | PSTRCR_120_RBP | MW358928.1 | QQK88320.1 | X21 | No |  |  |
| Group F | Uetakevirus | BUCT554_RBP | MW205203.1 | QQM14183.1 | X14 | No |  |  |
| Group F | Uetakevirus | F6699_Prophage_RBP | CP024266.1 | / | X7 | No |  |  |
| Group F | Uetakevirus | RHB30-C16_Prophage_RBP | CP057287.1 | / | O16 | HGT |  | 163 |
| Group F | Uetakevirus | SCU-116_Prophage_RBP | CP051719.1 | / | O2 | HGT |  | 206 |
| Group F | Vectrevirus | K1-5_RBP2 | NC_008152.1 | YP_654148.1 | K1 | RBP | (20) |  |
| Group F | Vectrevirus | K1-5_RBP1 | NC_008152.1 | YP_654147.1 | X16 | No |  |  |
| Group F | Vectrevirus | Mt1B1_P10_RBP2 | MT496971.1 | QNR52596.1 | X27 | No |  |  |

**Table S1:** Phage RBPs are first arranged according to their data set group, second to their phage genus and third to the RBP name. Serogroup specificity of the RBP is either confirmed at the RBP level (dark green), confirmed at the phage level (green) or the serogroup is dominant for at least 90 % of the RBPs within the RBP subtype (light green). When no serogroup was assigned for a RBP subtype, the receptor is listed as ‘X’, followed by a number for all RBPs within the same particular RBP subtype.

# References

1. Prokhorov NS, Riccio C, Zdorovenko EL, Shneider MM, Browning C, Knirel YA, et al. Function of bacteriophage G7C esterase tailspike in host cell adsorption. Mol Microbiol [Internet]. 2017 Aug 1 [cited 2022 Aug 10];105(3):385–98. Available from: https://onlinelibrary.wiley.com/doi/full/10.1111/mmi.13710

2. Bull JJ, Vimr ER, Molineux IJ. A tale of tails: Sialidase is key to success in a model of phage therapy against K1-capsulated Escherichia coli. Virology. 2010 Mar 1;398(1):79–86.

3. Scholl D, Merril C. The Genome of Bacteriophage K1F , a T7-Like Phage That Has Acquired the Ability To Replicate on K1 Strains of Escherichia coli †. Bacteriology. 2005;187(24):8499–503.

4. Plattner M, Shneider MM, Arbatsky NP, Shashkov AS, Chizhov AO, Nazarov S, et al. Structure and Function of the Branched Receptor-Binding Complex of Bacteriophage CBA120. J Mol Biol [Internet]. 2019;431(19):3718–39. Available from: http://dx.doi.org/10.1016/j.jmb.2019.07.022

5. Witte S, Zinsli L V., Gonzalez-Serrano R, Matter CI, Loessner MJ, van Mierlo JT, et al. Structural and functional characterization of the receptor binding proteins of Escherichia coli O157 phages EP75 and EP335. Comput Struct Biotechnol J. 2021 Jan 1;19:3416–26.

6. Barbirz S, Müller JJ, Uetrecht C, Clark AJ, Heinemann U, Seckler R. Crystal structure of Escherichia coli phage HK620 tailspike: Podoviral tailspike endoglycosidase modules are evolutionarily related. Mol Microbiol. 2008;69(2):303–16.

7. Scholl D, Gebhart D, Williams SR, Bates A, Mandrell R. Genome sequence of E. coli O104:H4 leads to rapid development of a targeted antimicrobial agent against this emerging pathogen. PLoS One. 2012;7(3):e33637.

8. Ritchie JM, Greenwich JL, Davis BM, Bronson RT, Gebhart D, Williams SR, et al. An Escherichia coli O157-Specific Engineered Pyocin Prevents and Ameliorates Infection by E. coli O157:H7 in an Animal Model of Diarrheal Disease. Antimicrob Agents Chemother [Internet]. 2011 Dec [cited 2022 Oct 14];55(12):5469. Available from: /pmc/articles/PMC3232761/

9. Liao Y Te, Salvador A, Harden LA, Liu F, Lavenburg VM, Li RW, et al. Characterization of a lytic bacteriophage as an antimicrobial agent for biocontrol of shiga toxin-producing escherichia coli o145 strains. Antibiotics. 2019;8(2).

10. Debarbieux LMD. Detection de souches E.coli de serotype o104. WO2013045863A1, 2012.

11. Zhang Y, Liao YT, Salvador A, Lavenburg VM, Wu VCH. Characterization of Two New Shiga Toxin-Producing Escherichia coli O103-Infecting Phages Isolated from an Organic Farm. Microorganisms [Internet]. 2021; Available from: https://doi.org/10.3390/microorganisms9071527

12. Liao YT, Liu F, Wu VCH. Complete Genome Sequence of a Lytic T7-Like Phage, Escherichia Phage vB_EcoP-Ro45lw, Isolated from Nonfecal Compost Samples. Microbiol Resour Announc [Internet]. 2019 Feb 28 [cited 2022 Oct 14];8(9). Available from: https://journals.asm.org/doi/10.1128/MRA.00036-19

13. Tsonos J, Oosterik LH, Tuntufye HN, Klumpp J, Butaye P, De Greve H, et al. A cocktail of in vitro efficient phages is not a guarantee for in vivo therapeutic results against avian colibacillosis. Vet Microbiol. 2014 Jul 16;171(3–4):470–9.

14. Sayers EW, Bolton EE, Brister JR, Canese K, Chan J, Comeau DC, et al. Database resources of the national center for biotechnology information. Nucleic Acids Res [Internet]. 2022 Jan 7 [cited 2023 Apr 13];50(D1):D20–6. Available from: https://pubmed.ncbi.nlm.nih.gov/34850941/

15. Pechacek D, Hwangbo M, Moreland R, Liu M, Ramsey J. Complete Genome Sequence of Escherichia coli Podophage Penshu1. Microbiol Resour Announc [Internet]. 2019 Sep 19 [cited 2022 Oct 14];8(38). Available from: /pmc/articles/PMC6753287/

16. Liu Y, Gong Q, Qian X, Li D, Zeng H, Li Y, et al. Prophage phiv205-1 facilitates biofilm formation and pathogenicity of avian pathogenic Escherichia coli strain DE205B. Vet Microbiol. 2020 Aug 1;247:108752.

17. Iversen H, L’Abée-Lund TM, Aspholm M, Arnesen LPS, Lindbäck T. Commensal E. coli Stx2 lysogens produce high levels of phages after spontaneous prophage induction. Front Cell Infect Microbiol. 2015;5(FEB):5.

18. Schwarzer D, Buettner FFR, Browning C, Nazarov S, Rabsch W, Bethe A, et al. A Multivalent Adsorption Apparatus Explains the Broad Host Range of Phage phi92: a Comprehensive Genomic and Structural Analysis. J Virol. 2012;86(19):10384–98.

19. Niu YD, McAllister TA, Nash JHE, Kropinski AM, Stanford K. Four Escherichia coli O157:H7 Phages: A New Bacteriophage Genus and Taxonomic Classification of T1-Like Phages. PLoS One [Internet]. 2014 Jun 25 [cited 2023 Apr 13];9(6):e100426. Available from: https://journals.plos.org/plosone/article?id=10.1371/journal.pone.0100426

20. Leiman PG, Battisti AJ, Bowman VD, Stummeyer K, Mühlenhoff M, Gerardy-Schahn R, et al. The Structures of Bacteriophages K1E and K1-5 Explain Processive Degradation of Polysaccharide Capsules and Evolution of New Host Specificities. J Mol Biol. 2007;371(3):836–49.
