# Supplementary material for: Phage tailspike modularity and horizontal gene transfer reveals specificity towards E. coli O-antigen serogroups
Source: Virol J. 2023 Aug 7;20:174. doi: 10.1186/s12985-023-02138-4 (PMC10408124; doi:10.1186/s12985-023-02138-4)
Supplement: Supplementary file 2 — Additional file 2. Table S2: E. coli strains encoding temperate phages belonging to genera Uetakevirus or Lederbergvirus. [file 12985_2023_2138_MOESM2_ESM.docx]

# Additional file 2

**Table S2: *E. coli* strains encoding temperate phages belonging to genera *Uetakevirus* or *Lederbergvirus*.**

| **Serogroup O26** | | | **Serogroup O103** | | | **Serogroup O104** | | | **Serogroup O111** | | |
| --- | --- | --- | --- | --- | --- | --- | --- | --- | --- | --- | --- |
| **Strain name** | **Accession** | **Prophage  inside** | **Strain name** | **Accession** | **Prophage inside** | **Strain name** | **Accession** | **Prophage inside** | **Strain name** | **Accession** | **Prophage inside** |
| 11368 | AP010953.1 | *Lederbergvirus* | 07-3866 | CP030781.2 | *Uetakevirus* | 2009EL-2050 | NC_018650.1 | *Lederbergvirus* | 11128 | NC_013364.1 | *Lederbergvirus* |
| 2013C-3996 | CP027572.1 | *Lederbergvirus* | **MINF_2E-sc-2280463** | LR890651.1 | *Uetakevirus* | 2009EL-2071 | NC_018661.1 | *Lederbergvirus* | **110512** | NZ_AP019761.1 | *Lederbergvirus* |
| 97-3250 | CP027599.1 | *Uetakevirus* | NCTC8196 | LR134270.1 | *Lederbergvirus* | 2011C-3493 | NC_018658.1 | *Lederbergvirus* | 2013C-3250 | CP027380.1 | *Lederbergvirus* |
| **E2865** | AP018808.1 | *Uetakevirus* | RHB17-C18 | CP057684.1 | *Lederbergvirus* | C227-11 | NZ_CP011331.1 | *Lederbergvirus* | 2013C-4081 | CP027573.1 | *Lederbergvirus* |
| FWSEC0001 | NZ_CP031922.1 | *Lederbergvirus* | RHB31-C15 | CP057259.1 | *Lederbergvirus* | FDAARGOS_348 | CP022086.2 | *Lederbergvirus* | E2863 | AP018802.1 | *Lederbergvirus* |
| **RM10386** | NZ_CP028126.1 | *Lederbergvirus* | **RHB38-C21** | CP057085.1 | *Lederbergvirus* | FDAARGOS_349 | NZ_CP027394.1 | *Lederbergvirus* | FDAARGOS_95 | CP014092.2 | *Lederbergvirus* |
|  |  |  | **RHBSTW-00777** | CP056165.1 | *Uetakevirus* | **LB226692** | NZ_CP024992.1 | *Lederbergvirus* | NCCP 14539 | CP042981.1 | *Lederbergvirus* |
|  |  |  | S56 | CP010242.1 | *Lederbergvirus* |  |  |  | SD134209 | CP029692.1 | *Lederbergvirus* |
| **Serogroup O145** | | | **Serogroup O146** | | | **Serogroup O157** | | |  |  |  |
| **Strain name** | **Accession** | **Prophage  inside** | **Strain name** | **Accession** | **Prophage inside** | **Strain name** | **Accession** | **Prophage inside** |  |  |  |
| CFSAN004176 | NZ_CP014583.1 | *Lederbergvirus* | **RHB38-C01** | CP057104.1 | *Lederbergvirus* | **TT12B** | CP038494.1 | *Uetakevirus* |  |  |  |
| CFSAN004177 | CP014670.1 | *Lederbergvirus* |  |  |  |  |  |  |  |  |  |
| **FHI58** | LM995999.1 | *Lederbergvirus* |  |  |  |  |  |  |  |  |  |
| FHI63 | LM996460.1 | *Lederbergvirus* |  |  |  |  |  |  |  |  |  |

**Table S2:** Chosen RBP subtype representatives are indicated in bold. A number of 26, 19, 32, 26, 22, 23, 14 and 39 *E. coli* genomes were screened for the respective serogroups O26, O91, O103, O104, O111, O145, O146 and O157. For serogroup O91, no prophages belonging to these groups were found.
