# Supplementary material for: Phage tailspike modularity and horizontal gene transfer reveals specificity towards E. coli O-antigen serogroups
Source: Virol J. 2023 Aug 7;20:174. doi: 10.1186/s12985-023-02138-4 (PMC10408124; doi:10.1186/s12985-023-02138-4)
Supplement: Supplementary file 3 — Additional file 3. Figures S1–S3: Similarity matrices of the RBP and its domains after MUSCLE multiple sequence alignment. [file 12985_2023_2138_MOESM3_ESM.pdf]

Additional file 3

Similarity matrices of the RBP and its domains after MUSCLE multiple sequence alignment.

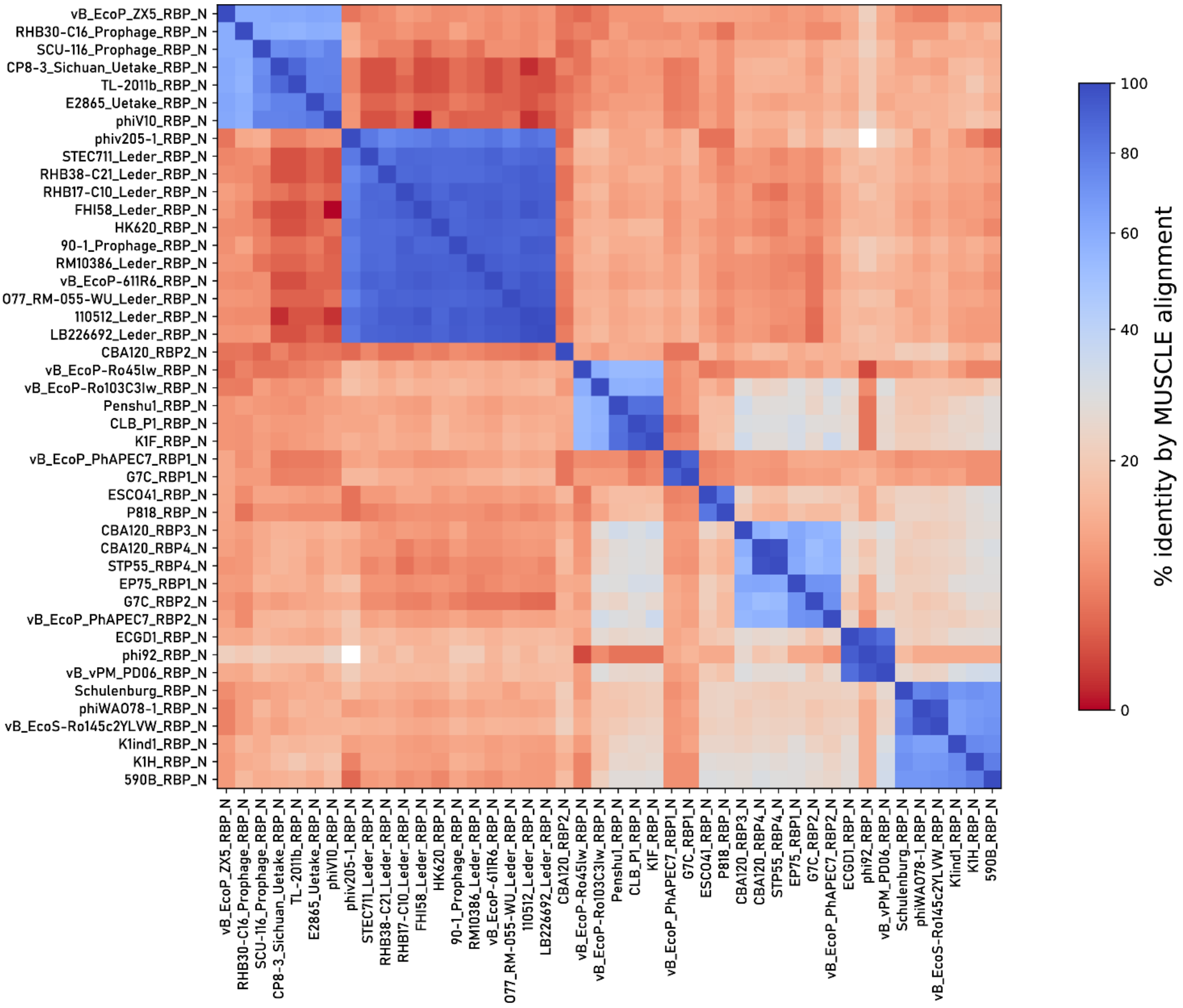

Figure S1: Similarity matrix of the RBP anchor domains after MUSCLE multiple sequence alignment. The % aa identity between all delineated anchor amino acid sequences is visualized. Clustering corresponds to the branching of the phylogenetic tree of the RBP anchor domains (**Figure 5**, **b**).

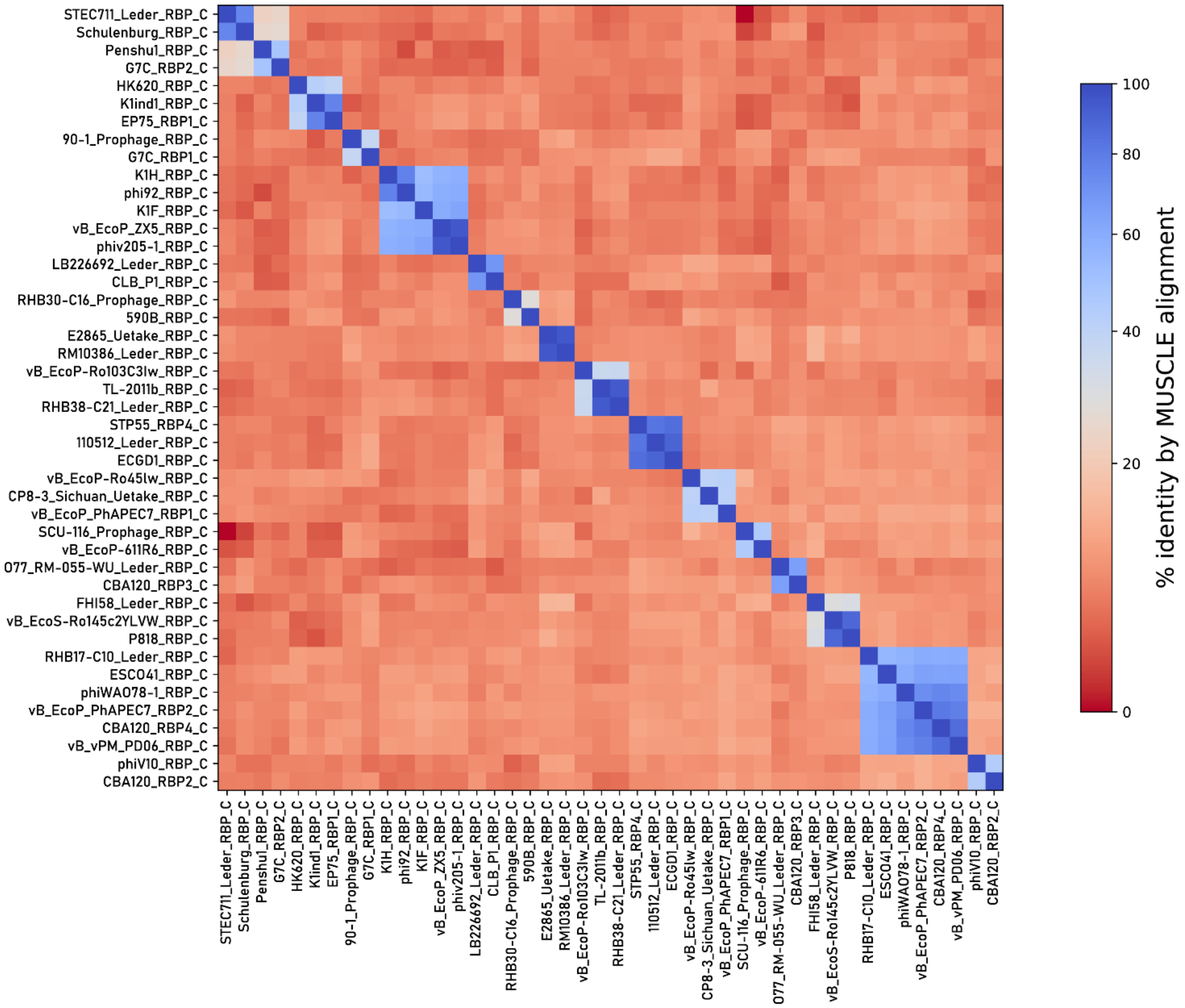

Figure S2: Similarity matrix of the RBP RBDs after MUSCLE multiple sequence alignment. The % aa identity between all delineated RBD amino acid sequences is visualized. Clustering corresponds to the branching of the phylogenetic tree of the RBDs (**Figure 5**, **c**).

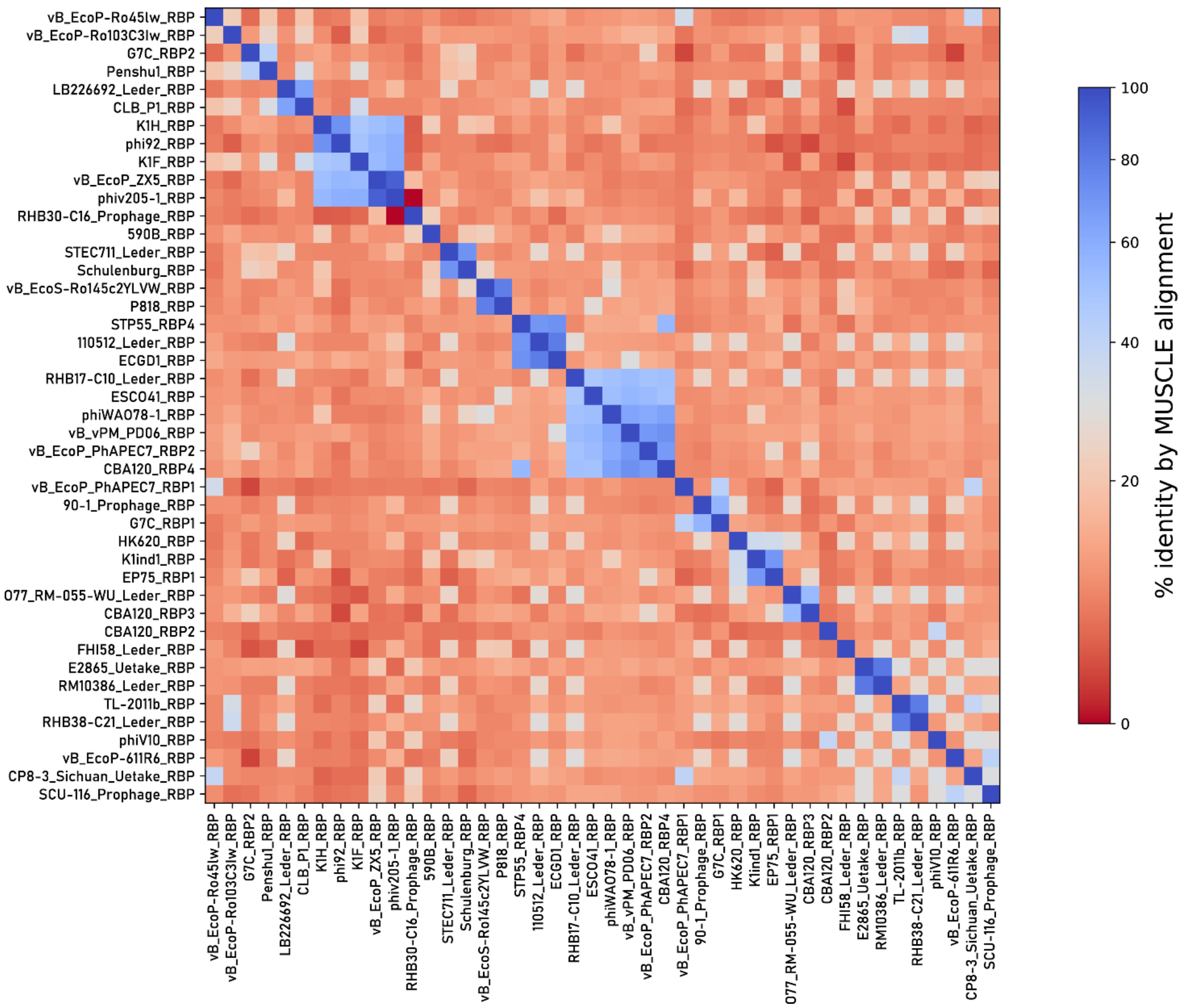

Figure S3: Similarity matrix of the complete RBPs after MUSCLE multiple sequence alignment. The % aa identity between all RBP amino acid sequences is visualized. Similarity matches correspond to the branching of the phylogenetic tree of the RBP coding sequences (**Figure 5**, d).
