# Supplementary material for: Phage tailspike modularity and horizontal gene transfer reveals specificity towards E. coli O-antigen serogroups
Source: Virol J. 2023 Aug 7;20:174. doi: 10.1186/s12985-023-02138-4 (PMC10408124; doi:10.1186/s12985-023-02138-4)
Supplement: Supplementary file 4 — Additional file 4. Figure S4: Predicted quaternary structures of the phage RBPs using AlphaFold2. [file 12985_2023_2138_MOESM4_ESM.pdf]

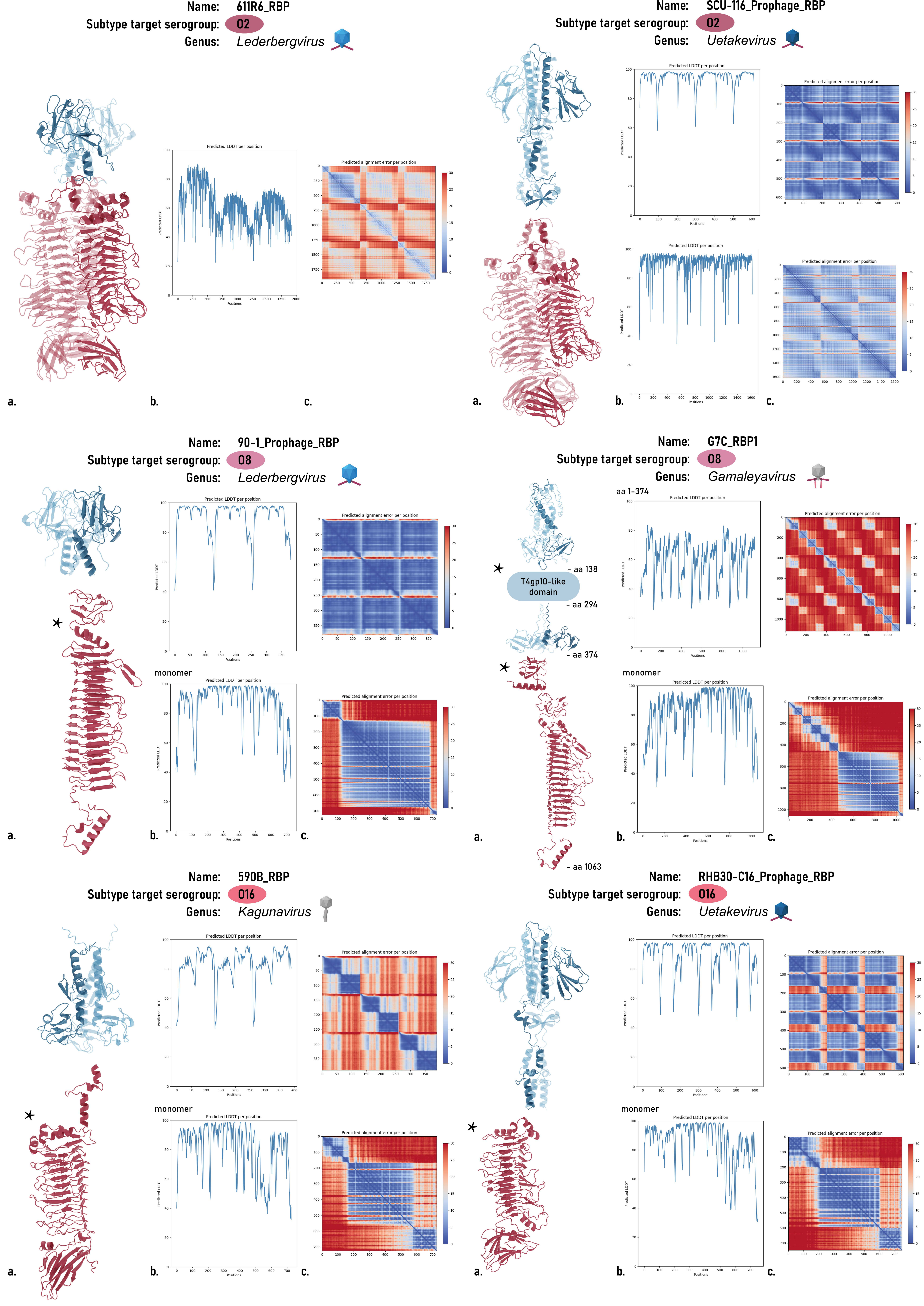

Name: 611R6_RBP
Subtype target serogroup: O2
Genus: Lederbergvirus
Predicted LDDT per position
Predicted LDDT
Positions
Predicted alignment error per position
a.
b.
c.
Name: SCU-116_Prophage_RBP
Subtype target serogroup: O2
Genus: Uetakevirus
Predicted LDDT per position
Predicted alignment error per position
a.
b.
c.
Name: 90-1_Prophage_RBP
Subtype target serogroup: O8
Genus: Lederbergvirus
monomer
Predicted LDDT per position
Predicted alignment error per position
a.
b.
c.
Name: G7C_RBP1
Subtype target serogroup: O8
Genus: Gamaleyavirus
aa 1-374
- aa 138
T4gp10-like domain
- aa 294
- aa 374
- aa 1063
monomer
Predicted LDDT per position
Predicted alignment error per position
a.
b.
c.
Name: 590B_RBP
Subtype target serogroup: O16
Genus: Kagunavirus
monomer
Predicted LDDT per position
Predicted alignment error per position
a.
b.
c.
Name: RHB30-C16_Prophage_RBP
Subtype target serogroup: O16
Genus: Uetakevirus
monomer
Predicted LDDT per position
Predicted alignment error per position
a.
b.
c.

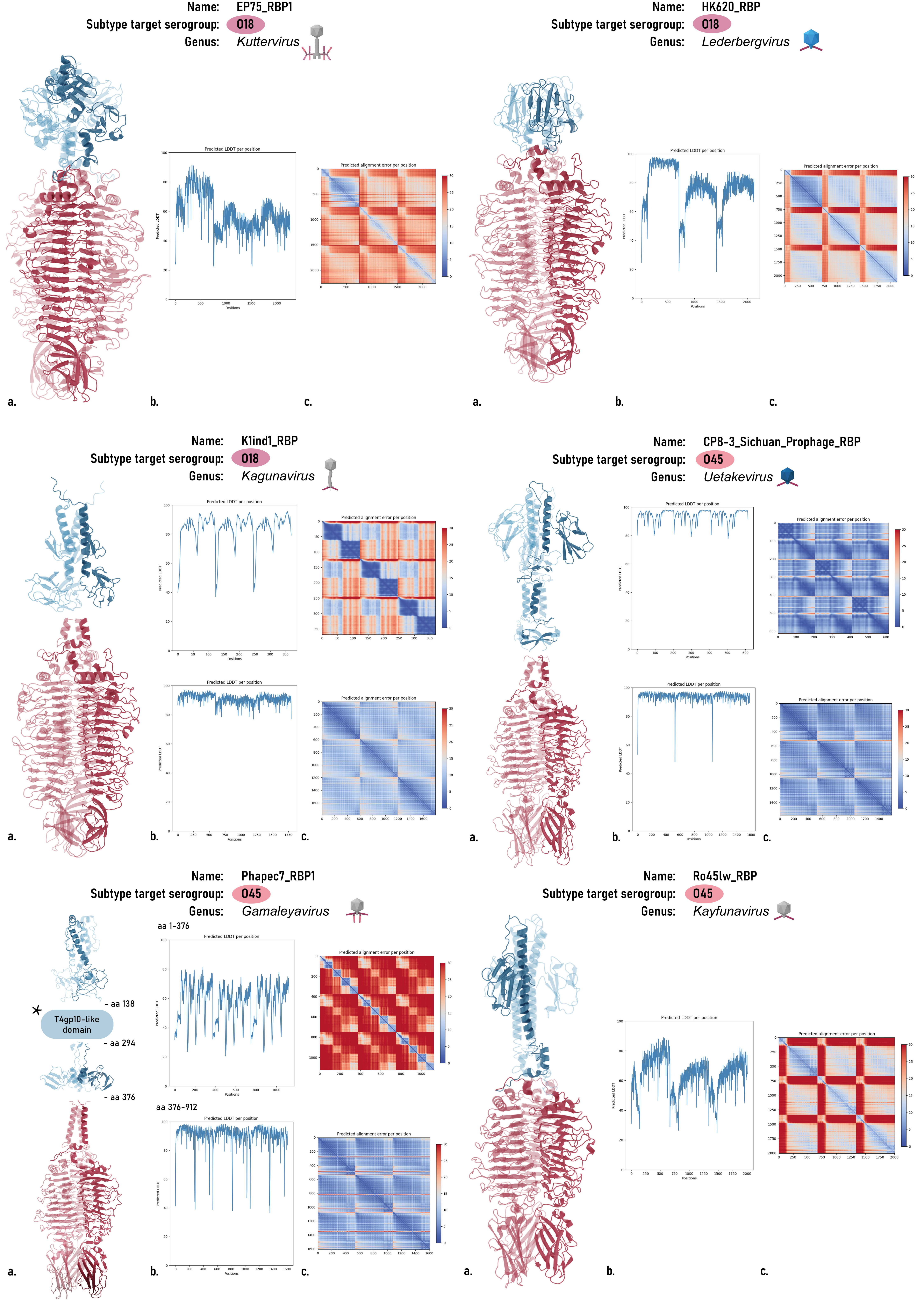

Name: EP75_RBP1
Subtype target serogroup: O18
Genus: Kuttervirus
Predicted LDDT per position
Predicted alignment error per position
Positions
Predicted LDDT
a.
b.
c.
Name: HK620_RBP
Subtype target serogroup: O18
Genus: Lederbergvirus
Predicted LDDT per position
Predicted alignment error per position
Positions
Predicted LDDT
a.
b.
c.
Name: K1ind1_RBP
Subtype target serogroup: O18
Genus: Kagunavirus
Predicted LDDT per position
Predicted alignment error per position
Positions
Predicted LDDT
a.
b.
c.
Name: CP8-3_Sichuan_Prophage_RBP
Subtype target serogroup: O45
Genus: Uetakevirus
Predicted LDDT per position
Predicted alignment error per position
Positions
Predicted LDDT
a.
b.
c.
Name: Phapec7_RBP1
Subtype target serogroup: O45
Genus: Gamaleyavirus
aa 1-376
aa 376-912
- aa 138
T4gp10-like domain
- aa 294
- aa 376
Predicted LDDT per position
Predicted alignment error per position
Positions
Predicted LDDT
a.
b.
c.
Name: Ro45lw_RBP
Subtype target serogroup: O45
Genus: Kayfunavirus
Predicted LDDT per position
Predicted alignment error per position
Positions
Predicted LDDT
a.
b.
c.

Name: E2865_Prophage_RBP
Subtype target serogroup: O26
Genus: *Uetakevirus*

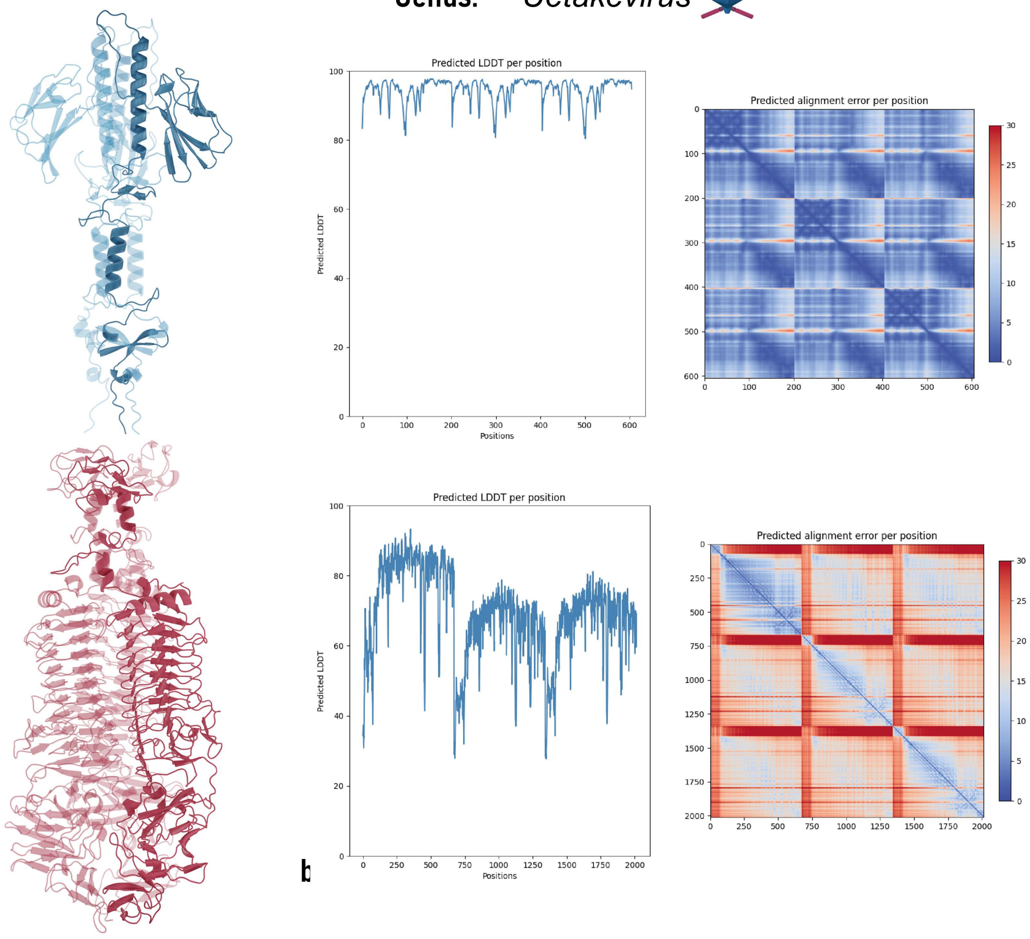

Name: RM10386_Prophage_RBP
Subtype target serogroup: O26
Genus: *Lederbergvirus*

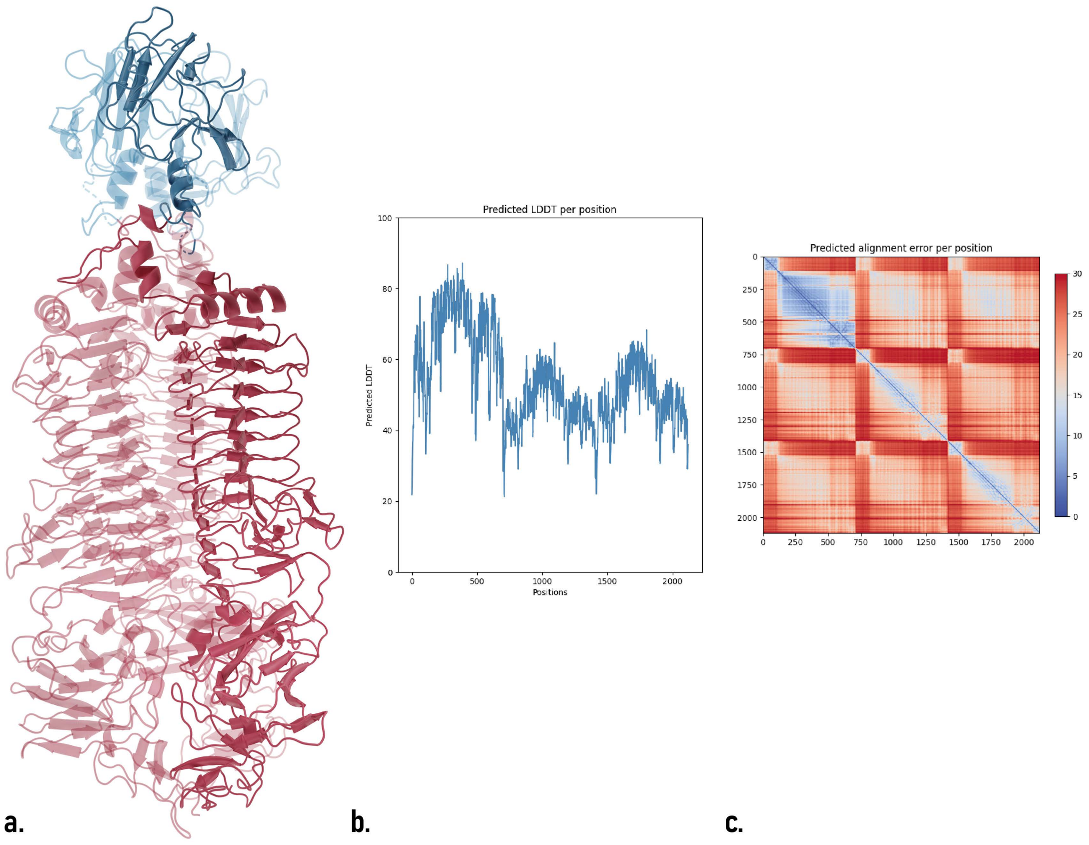

Name: CBA120_RBP3
Subtype target serogroup: O77
Genus: *Kuttervirus*

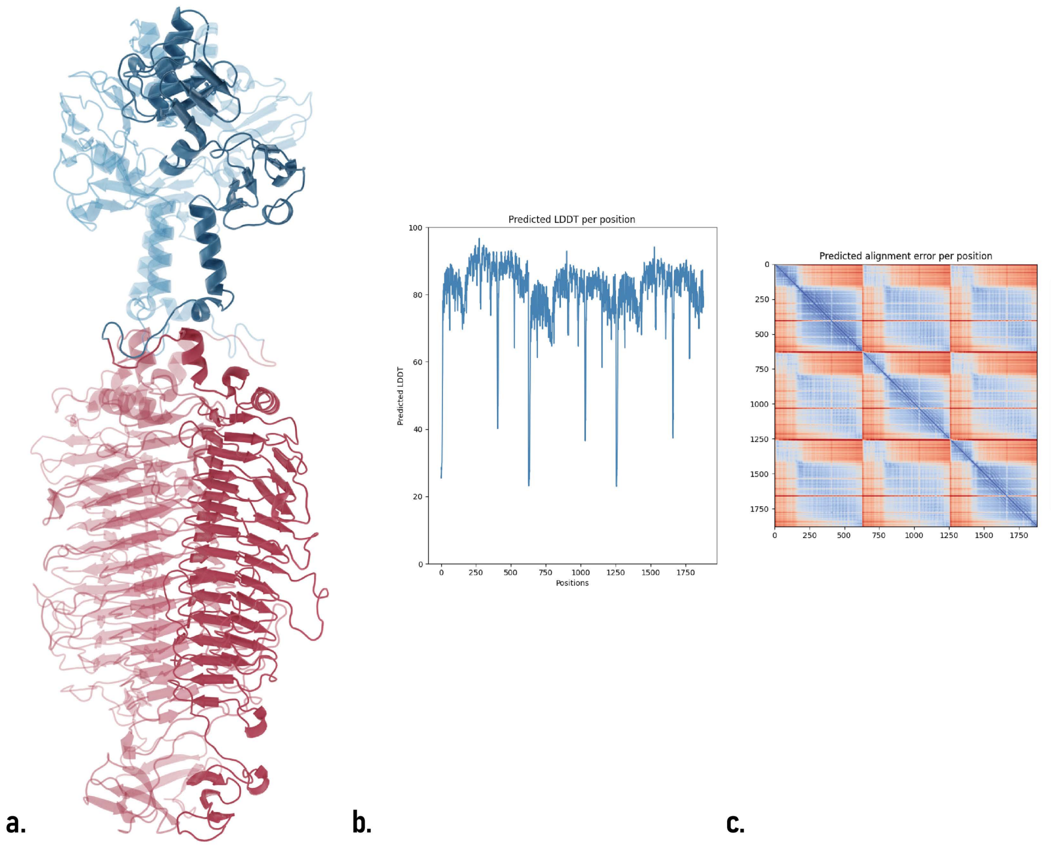

Name: RM-055-WU_Prophage_RBP
Subtype target serogroup: O77
Genus: *Lederbergvirus*

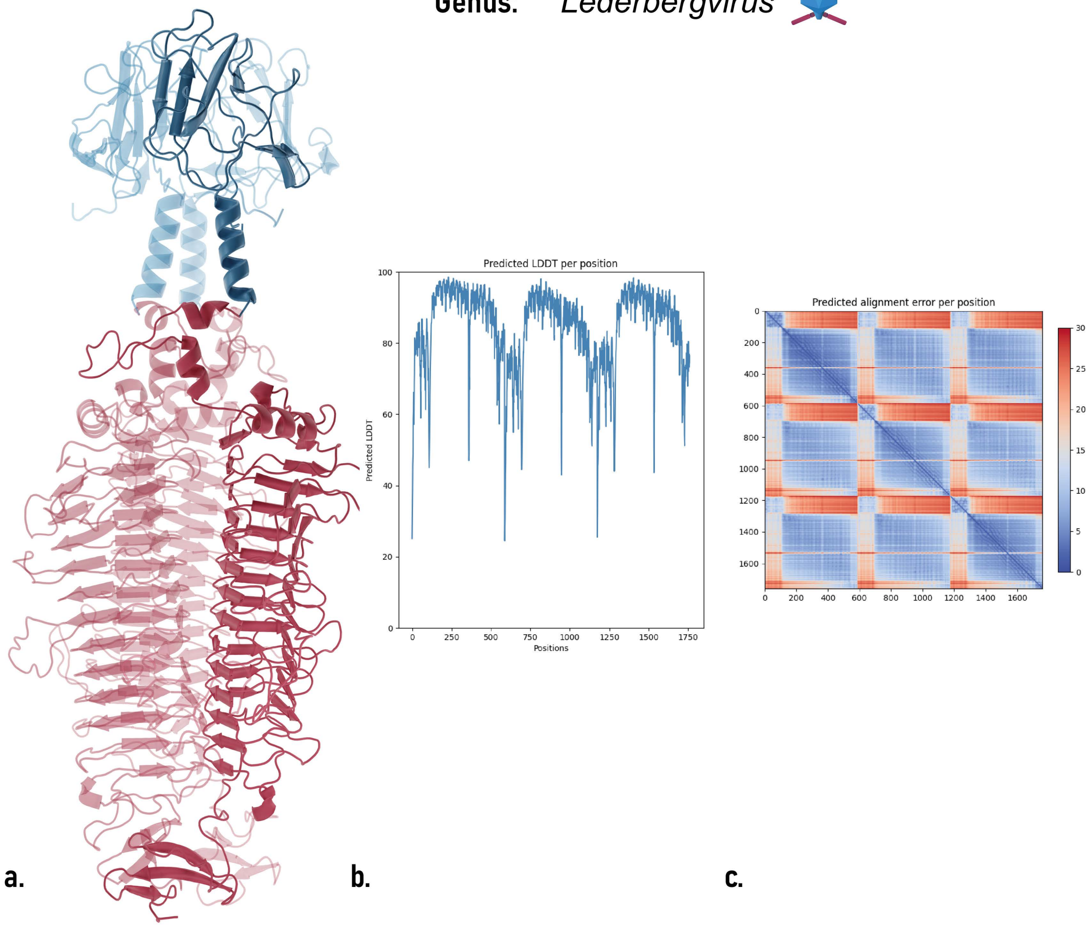

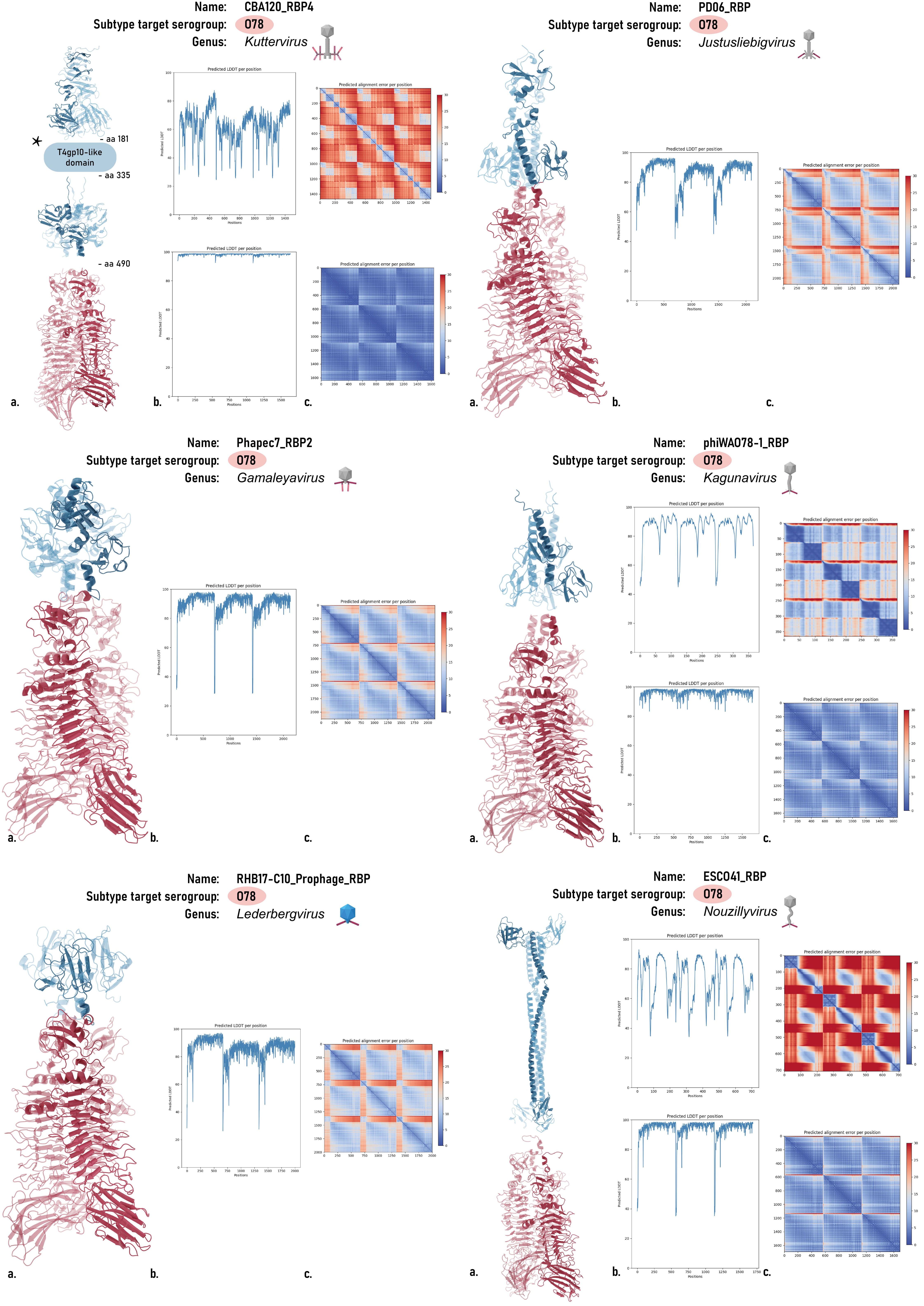

Name: CBA120_RBP4
Subtype target serogroup: O78
Genus: *Kuttervirus*
aa 181
T4gp10-like domain
aa 335
aa 490
Predicted LDDT per position
Predicted alignment error per position
Positions
Predicted LDDT
a.
b.
c.
Name: PD06_RBP
Subtype target serogroup: O78
Genus: *Justusliebigvirus*
a.
b.
c.
Name: Phapec7_RBP2
Subtype target serogroup: O78
Genus: *Gamaleyavirus*
a.
b.
c.
Name: phiWAO78-1_RBP
Subtype target serogroup: O78
Genus: *Kagunavirus*
a.
b.
c.
Name: RHB17-C10_Prophage_RBP
Subtype target serogroup: O78
Genus: *Lederbergvirus*
a.
b.
c.
Name: ESCO41_RBP
Subtype target serogroup: O78
Genus: *Nouzillyvirus*
a.
b.
c.

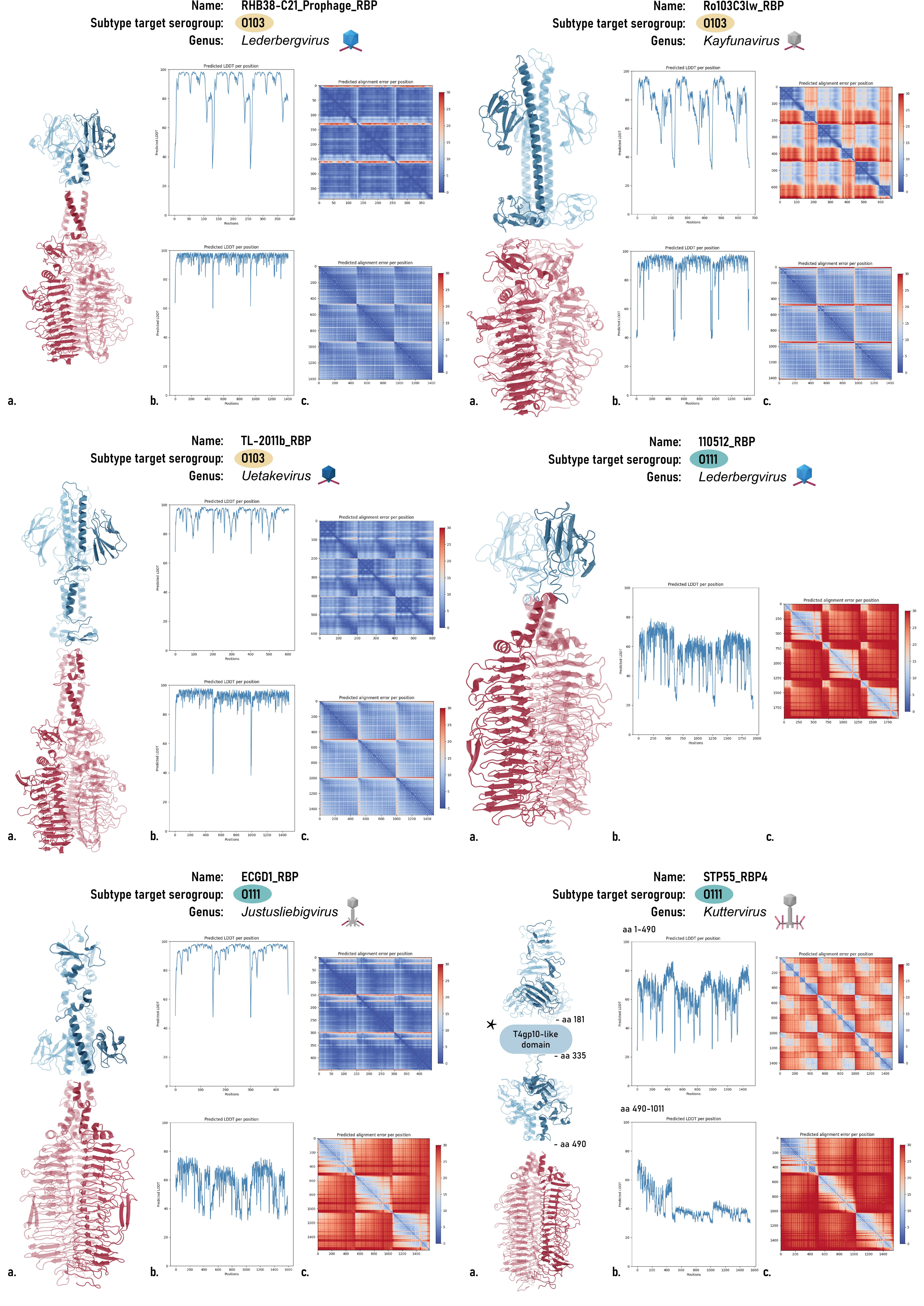
Name: RHB38-C21_Prophage_RBP
Subtype target serogroup: O103
Genus: Lederbergvirus
Name: Ro103C3lw_RBP
Subtype target serogroup: O103
Genus: Kayfunavirus
Predicted LDDT per position
Predicted alignment error per position
Positions
Predicted LDDT
a.
b.
c.
Name: TL-2011b_RBP
Subtype target serogroup: O103
Genus: Uetakevirus
Name: 110512_RBP
Subtype target serogroup: O111
Genus: Lederbergvirus
Name: ECGD1_RBP
Subtype target serogroup: O111
Genus: Justusliebigvirus
Name: STP55_RBP4
Subtype target serogroup: O111
Genus: Kuttervirus
aa 1-490
aa 490-1011
- aa 181
T4gp10-like domain
- aa 335
- aa 490

Name: CLB-P1_RBP
Subtype target serogroup: O104
Genus: *Kayfunavirus*

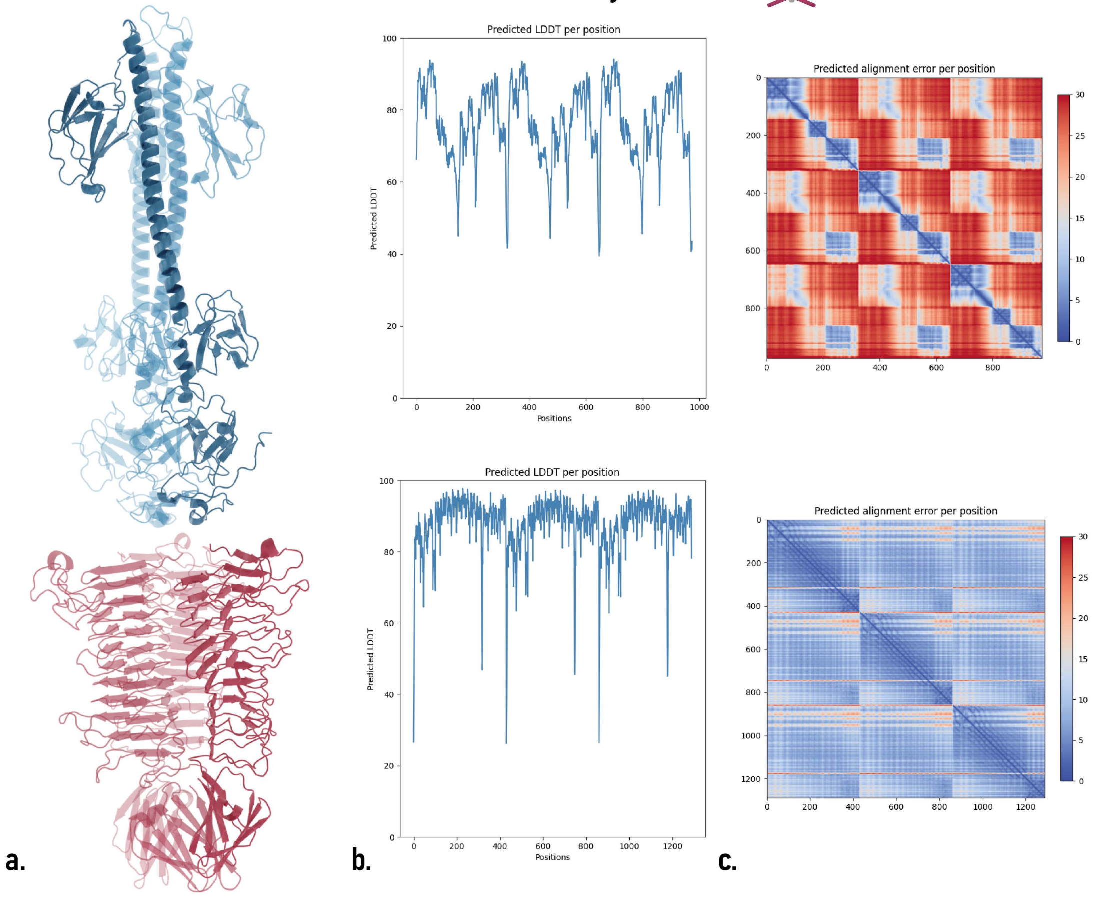

Name: LB226692_Prophage_RBP
Subtype target serogroup: O104
Genus: *Lederbergvirus*

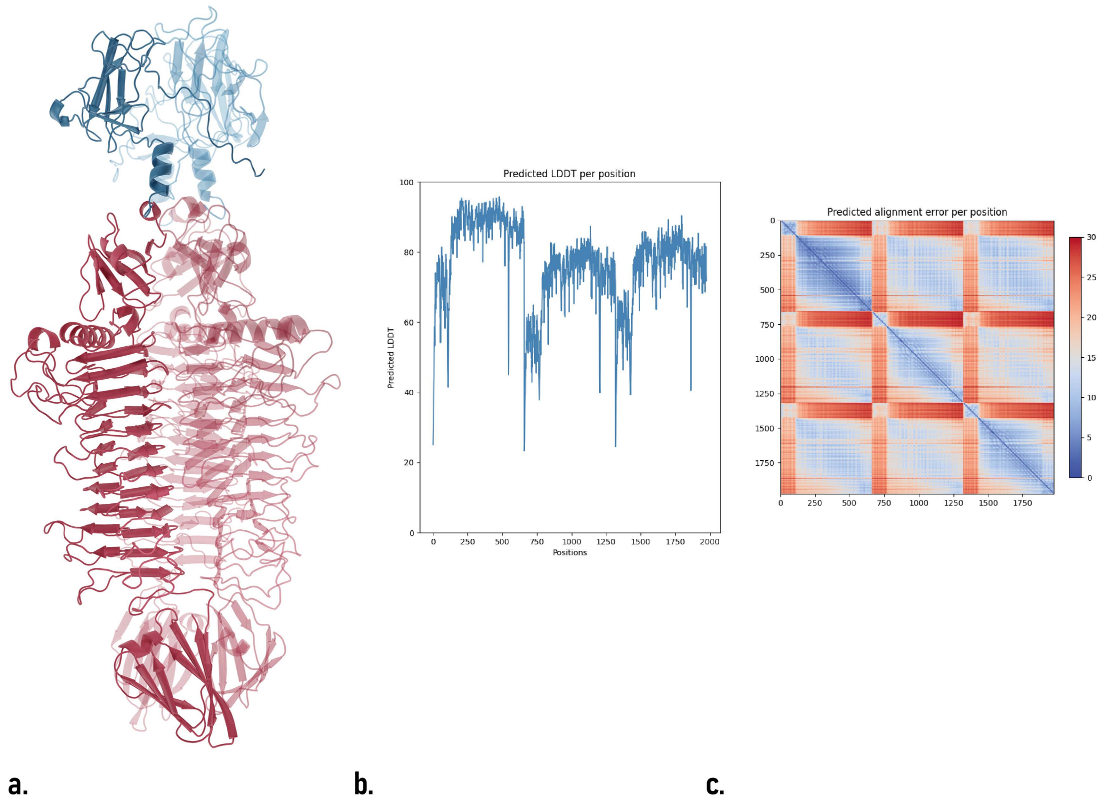

Name: FHI58_Prophage_RBP
Subtype target serogroup: O145
Genus: *Lederbergvirus*

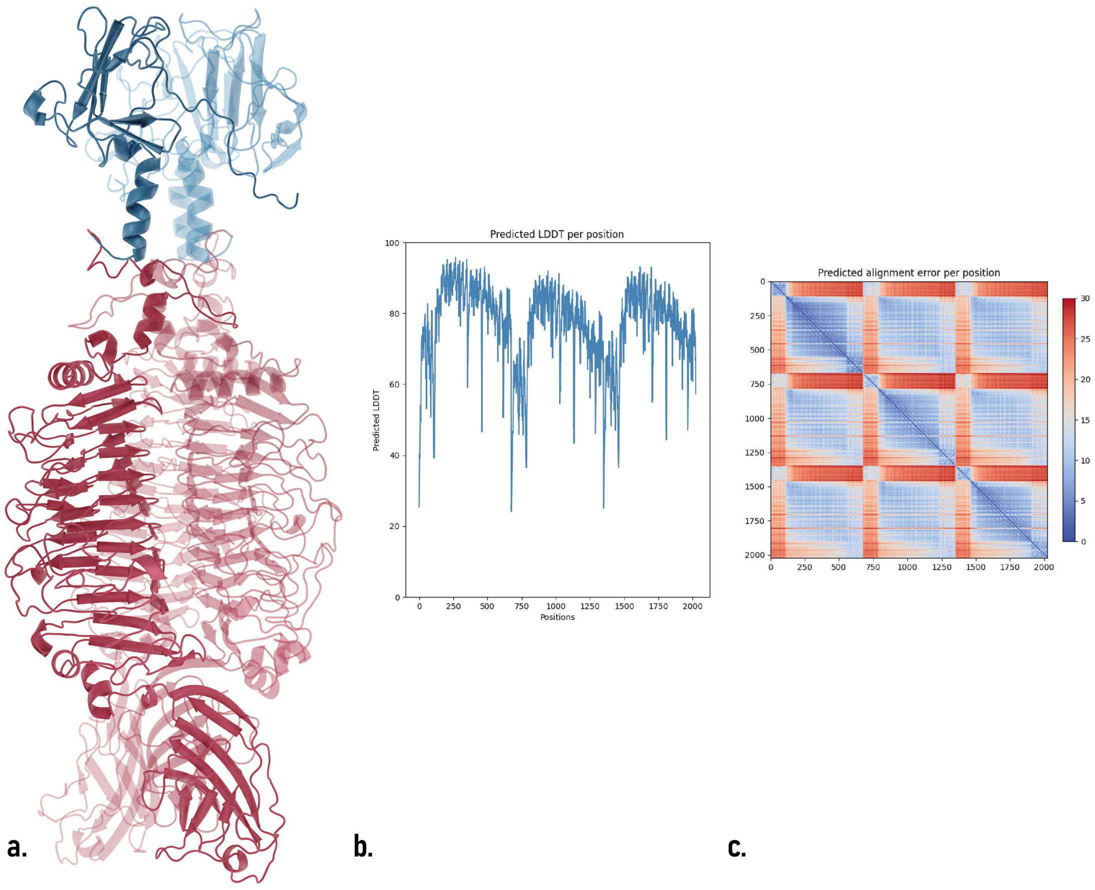

Name: Ro145c2YLVW_RBP
Subtype target serogroup: O145
Genus: *Kagunavirus*

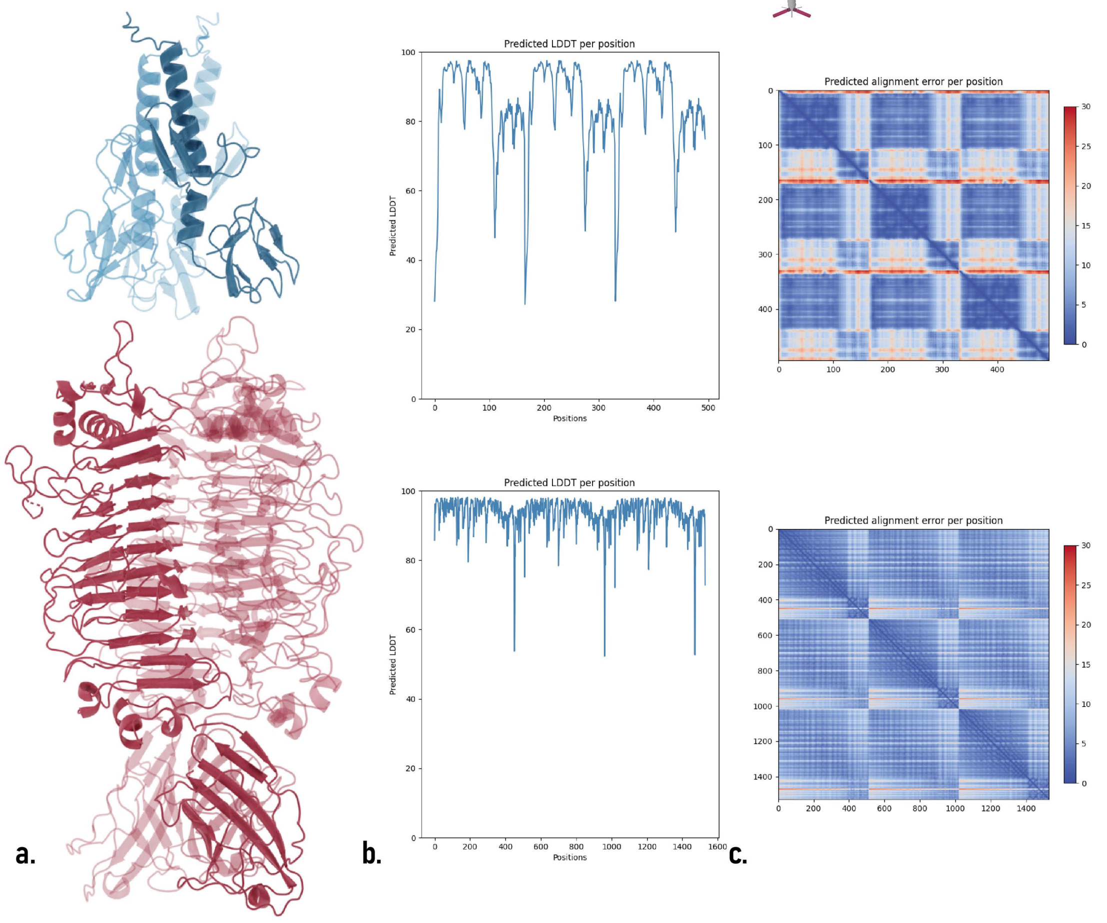

Name: P818_RBP
Subtype target serogroup: O145
Genus: *Nouzillyvirus*

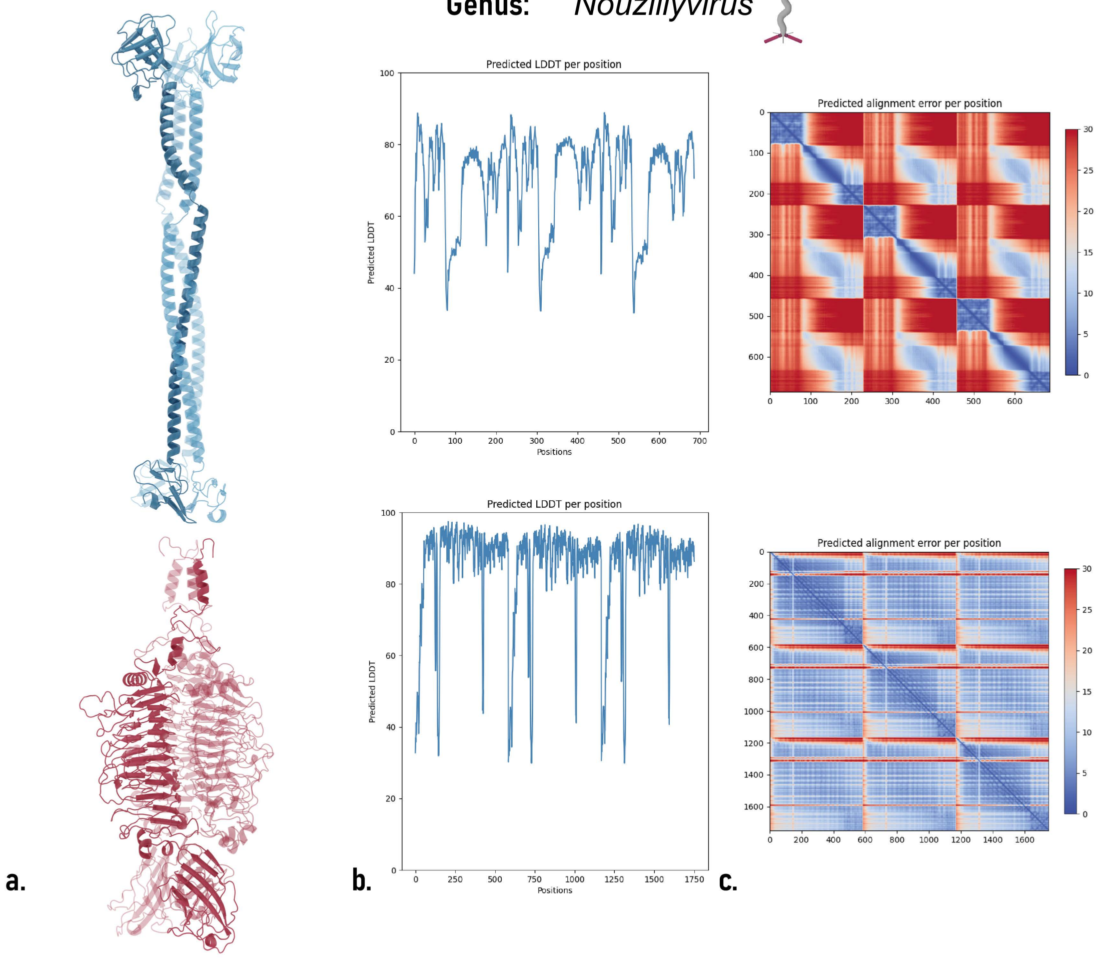

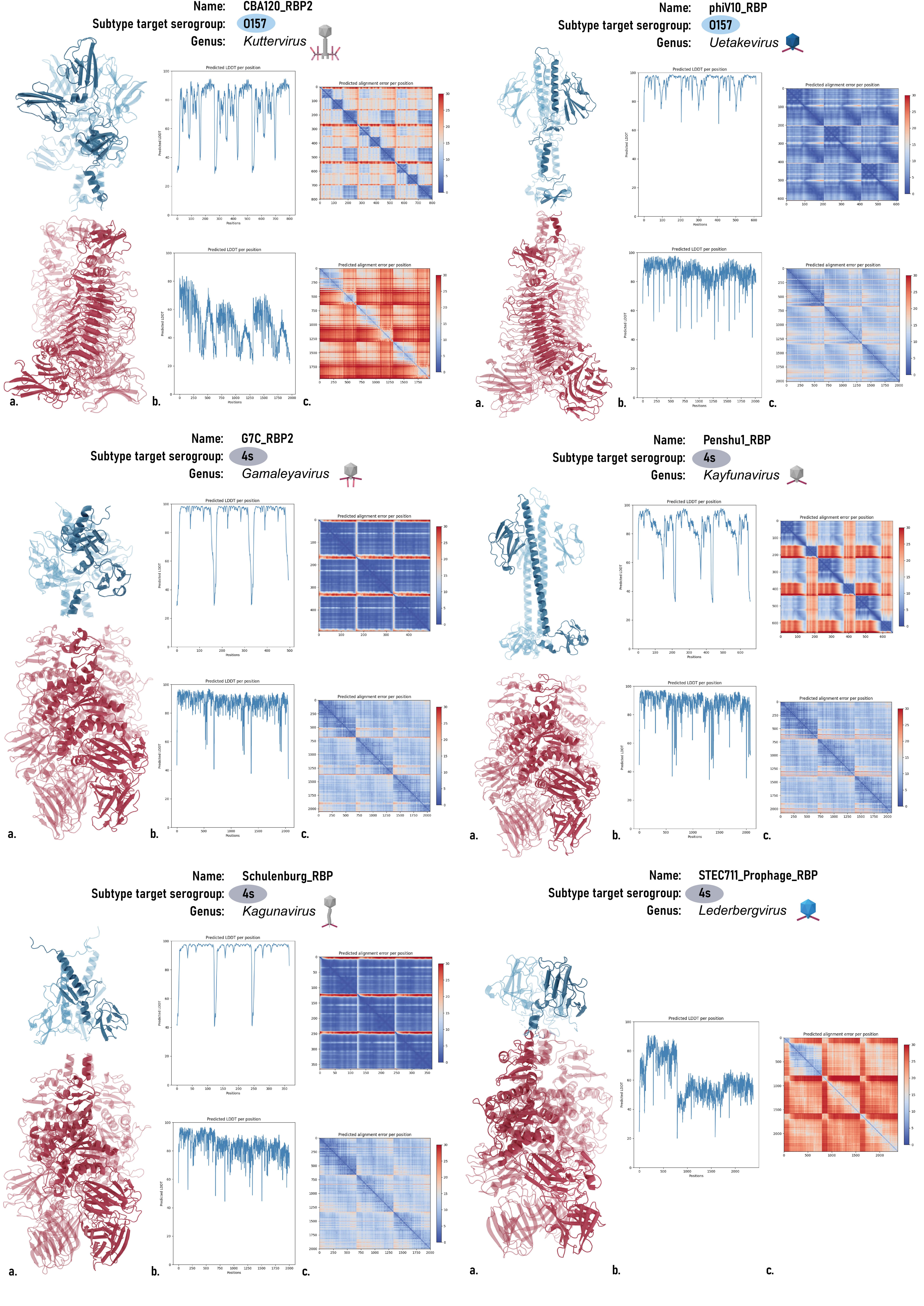

Name: CBA120_RBP2
Subtype target serogroup: O157
Genus: Kuttervirus
Name: phiV10_RBP
Subtype target serogroup: O157
Genus: Uetakevirus
Name: G7C_RBP2
Subtype target serogroup: 4s
Genus: Gamaleyavirus
Name: Penshu1_RBP
Subtype target serogroup: 4s
Genus: Kayfunavirus
Name: Schulenburg_RBP
Subtype target serogroup: 4s
Genus: Kagunavirus
Name: STEC711_Prophage_RBP
Subtype target serogroup: 4s
Genus: Lederbergvirus
Predicted LDDT per position
Predicted alignment error per position
Positions
Predicted LDDT
a.
b.
c.

Name: K1H_RBP
Subtype target serogroup: K1
Genus: *Kagunavirus*

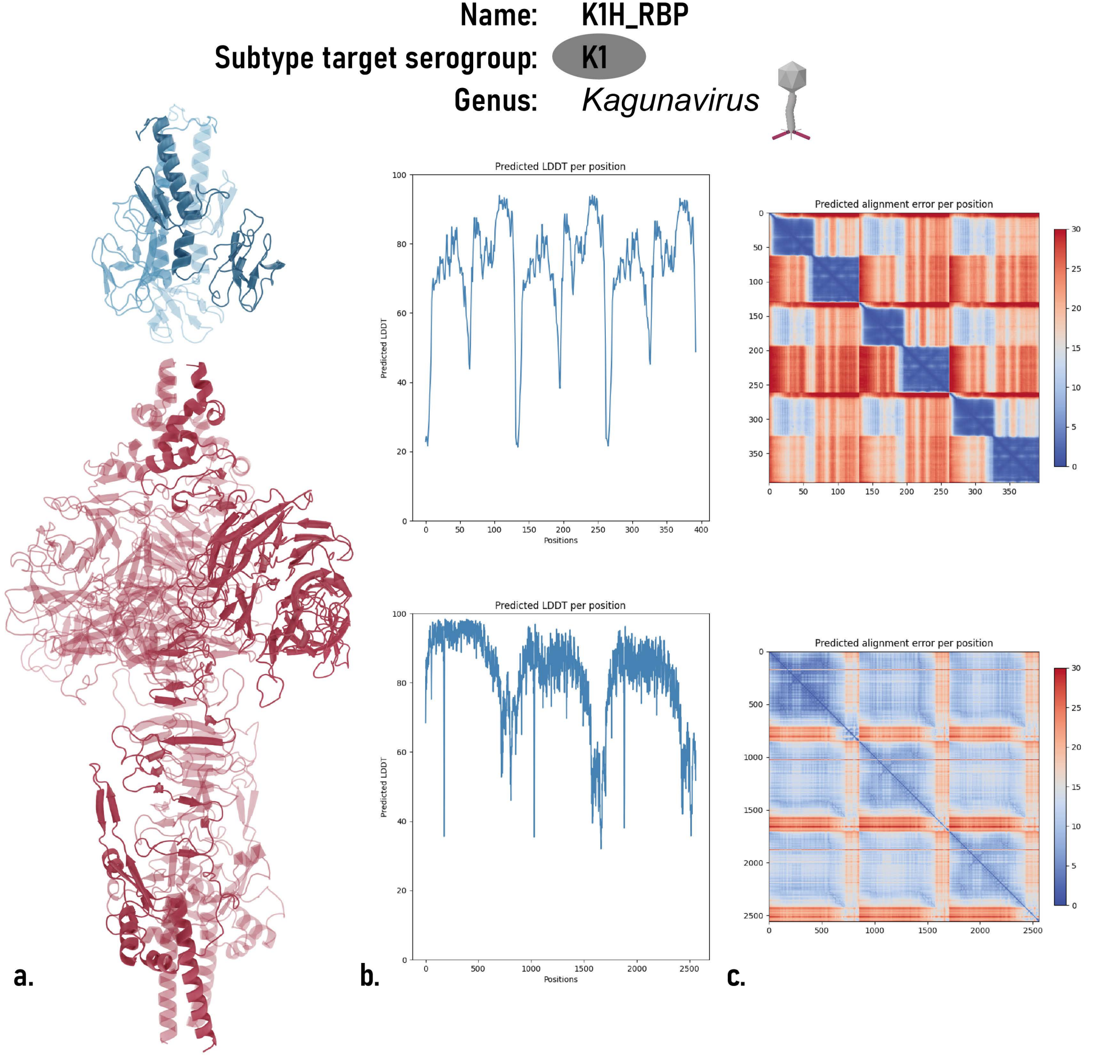

Name: K1F_RBP
Subtype target serogroup: K1
Genus: *Kayfunavirus*

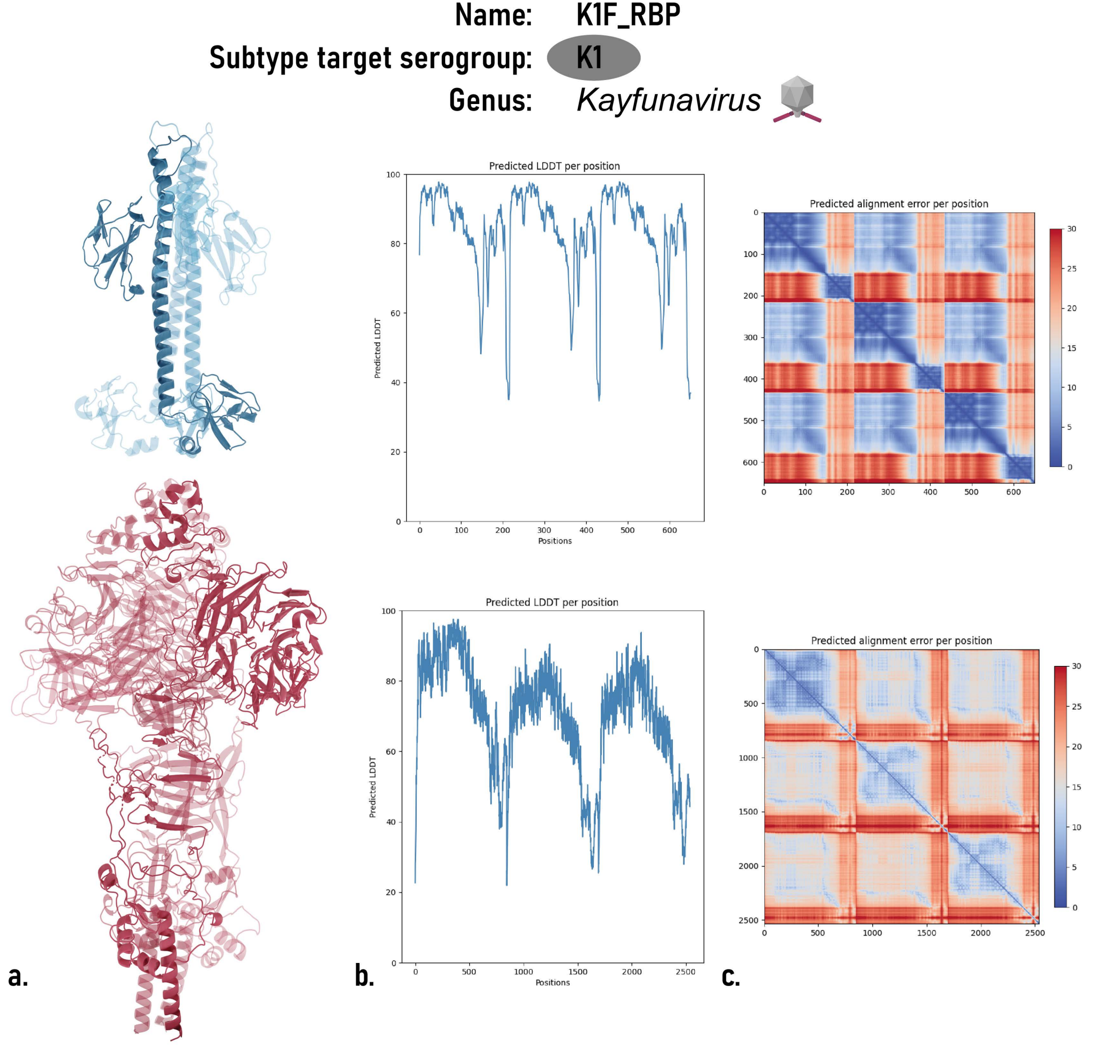

Name: Phi92_RBP
Subtype target serogroup: K1
Genus: *Justusliebigvirus*

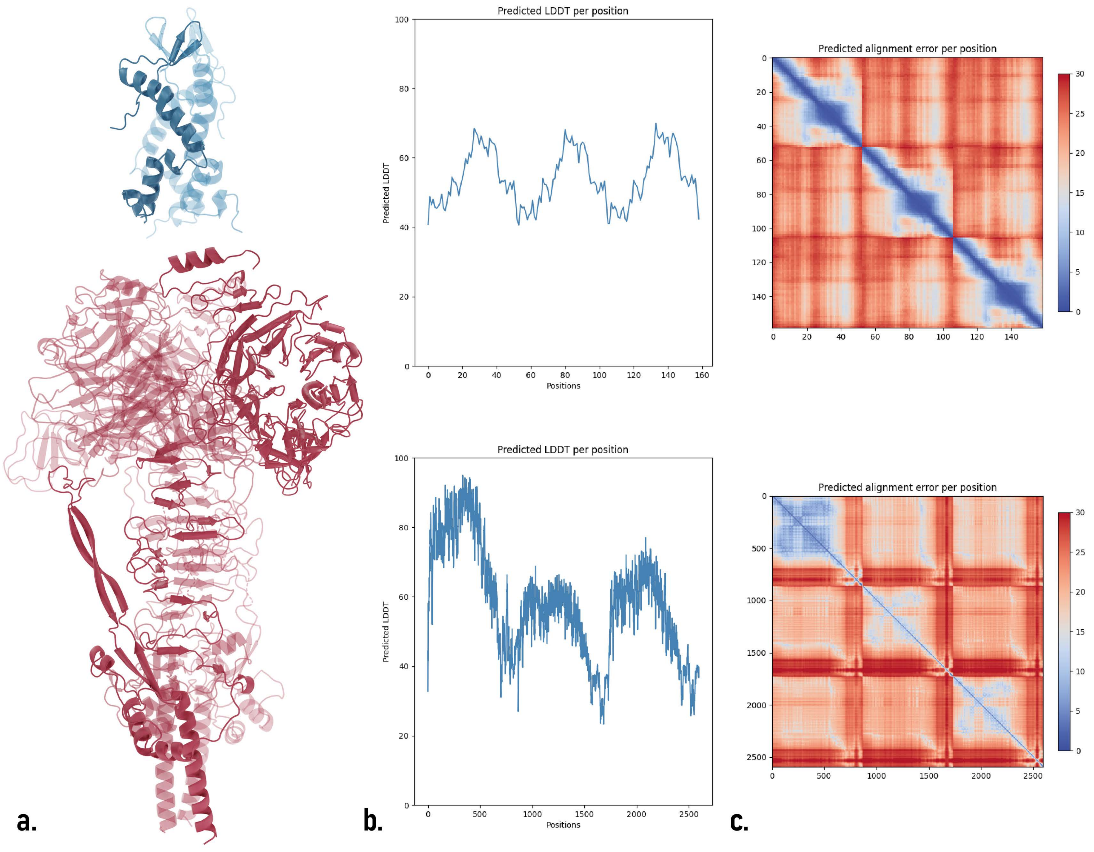

Name: Phiv205-1_RBP
Subtype target serogroup: K1
Genus: *Lederbergvirus*

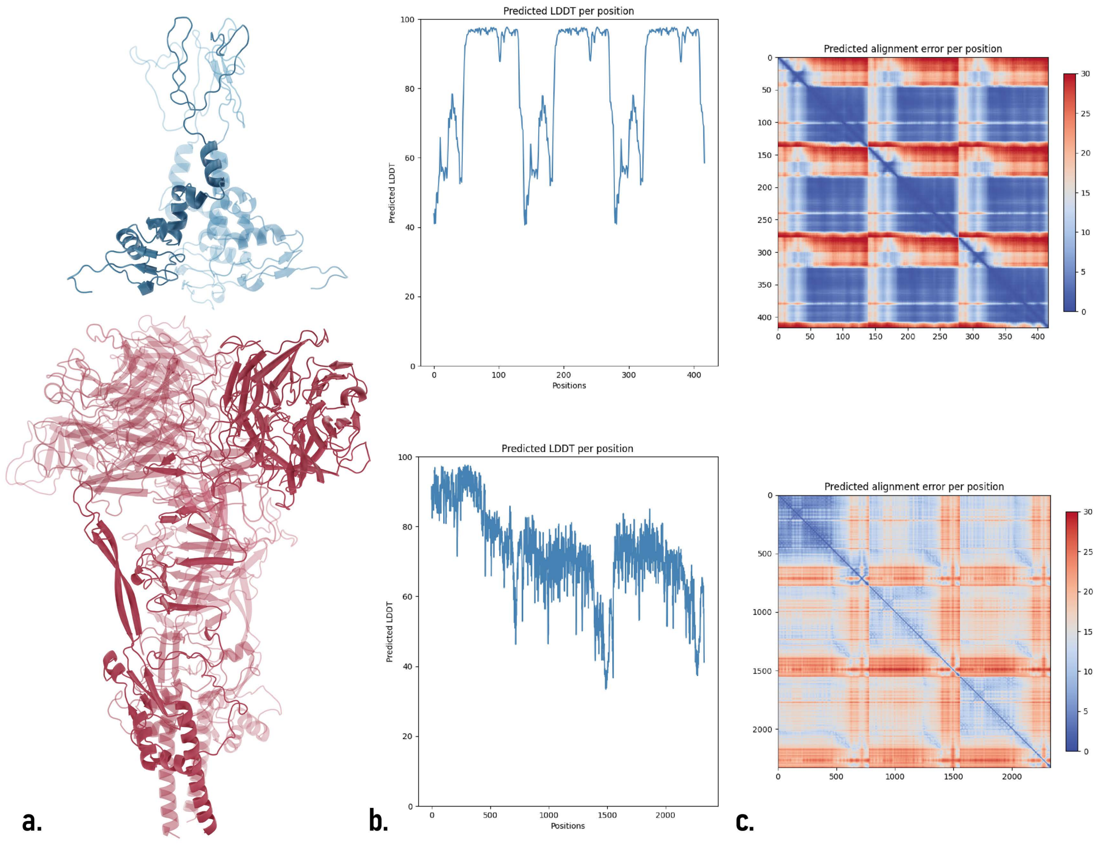

Name: ZX5_RBP
Subtype target serogroup: K1
Genus: *Uetakevirus*

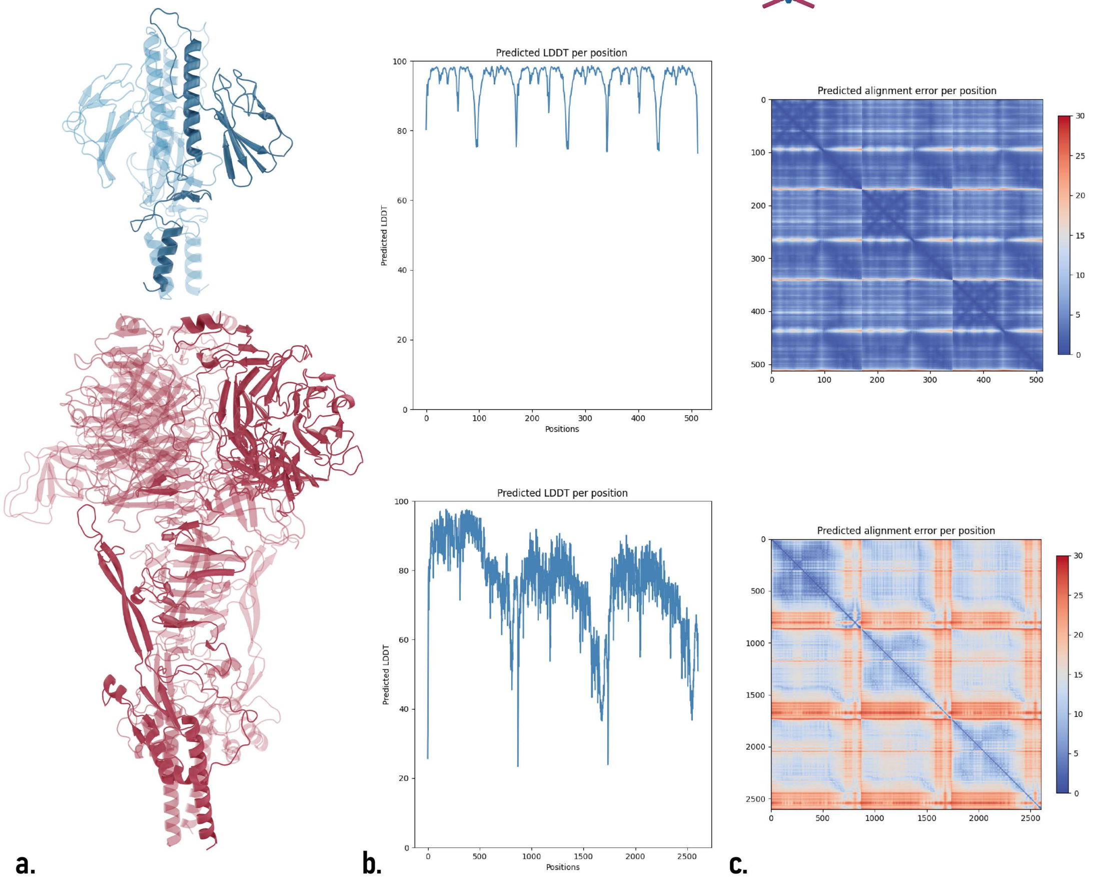

Additional file 4: Figure S4: **Predicted quaternary structures of the phage RBPs using AlphaFold2.** RBPs are clustered according to RBP subtype (a) Anchor domain shown in blue and RBD indicated in red, using different shades for each of the three monomers. An asterisk was used to indicate when quaternary structure predictions of RBP domains resulted in erroneous structures. (b) AlphaFold2 predicted LDDT per amino acid position; the upper graph represents the anchor domain and the lower graph the RBD when the structure was predicted separately. (c) AlphaFold2 predicted alignment error per amino acid position; the upper graph represents the anchor domain and the lower graph the RBD when the structure was predicted separately. When quaternary structure predictions of domains were unsuccessful, the tertiary structure was displayed instead.
