# Supplementary material for: Phage tailspike modularity and horizontal gene transfer reveals specificity towards E. coli O-antigen serogroups
Source: Virol J. 2023 Aug 7;20:174. doi: 10.1186/s12985-023-02138-4 (PMC10408124; doi:10.1186/s12985-023-02138-4)
Supplement: Supplementary file 5 — Additional file 5. Figure S5: DNA sequence motifs surrounding the RBD show higher sequence conservation than surrounding regions. [file 12985_2023_2138_MOESM5_ESM.pdf]

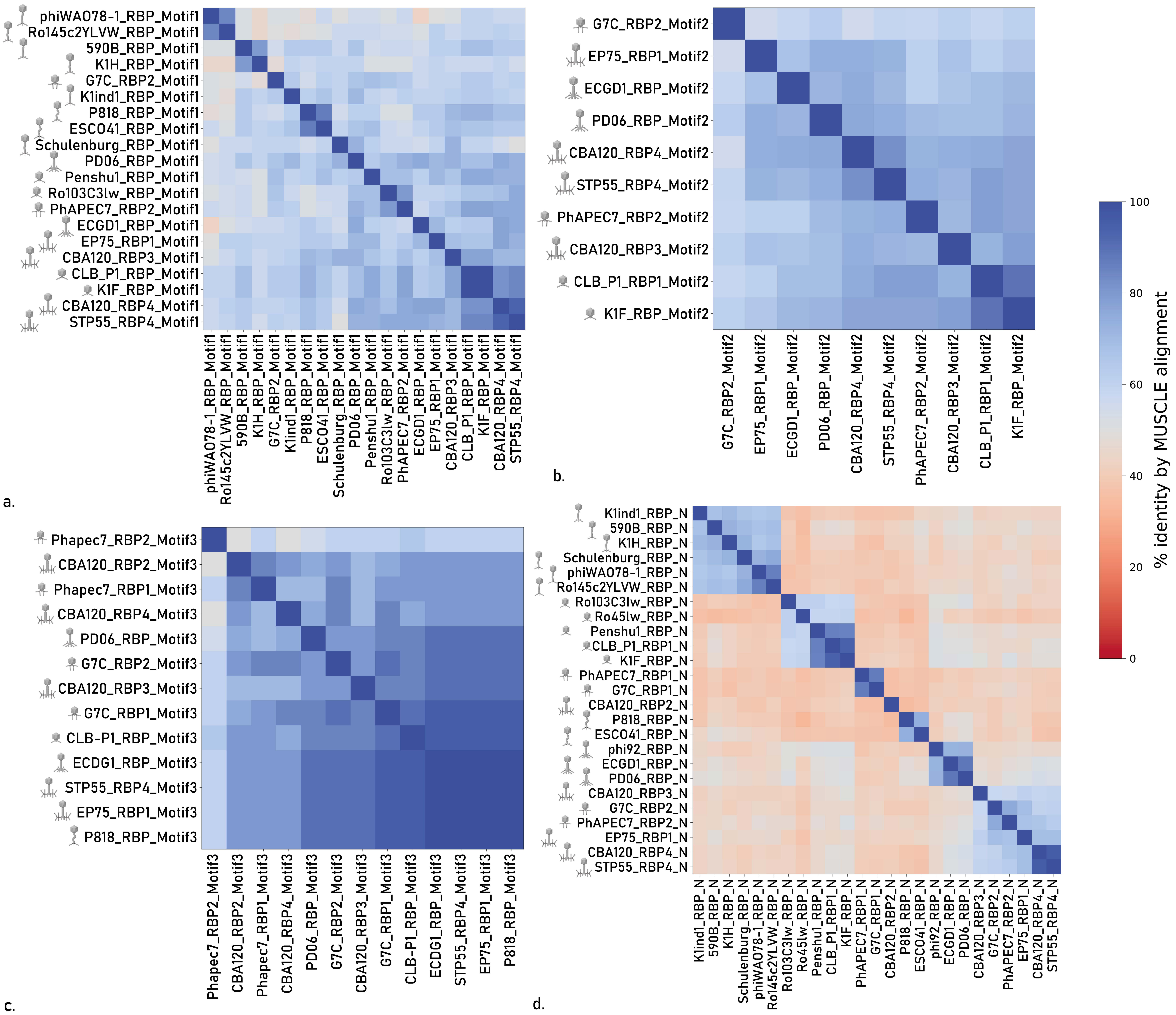

Additional file 5: Figure S5: DNA sequence motifs surrounding the RBD show higher sequence conservation than surrounding regions. DNA sequence % identity plots of three identified motifs (a-c) in the RBP regions of lytic phages belonging to different genera within the final data set. Conserved motifs with respective sample sizes 20, 10 and 13 and DNA sequence lengths 44, 95 and 20 nt indicate homologous DNA sequence regions surrounding the RBD conserved across the different lytic phage genera. (d) Control DNA sequence identity matrix of the DNA sequence of the N-terminal anchor domains of the RBPs containing the sequences of motif one and two.
