# Supplementary material for: Phage tailspike modularity and horizontal gene transfer reveals specificity towards E. coli O-antigen serogroups
Source: Virol J. 2023 Aug 7;20:174. doi: 10.1186/s12985-023-02138-4 (PMC10408124; doi:10.1186/s12985-023-02138-4)
Supplement: Supplementary file 6 — Additional file 6. Figures S6–S8: Multiple sequence alignments of the motif DNA sequences of the RBPs of phages in the final data set. [file 12985_2023_2138_MOESM6_ESM.pdf]

Additional file 6

Multiple sequence alignments of the motif DNA sequences of the RBPs of phages in the final data set.

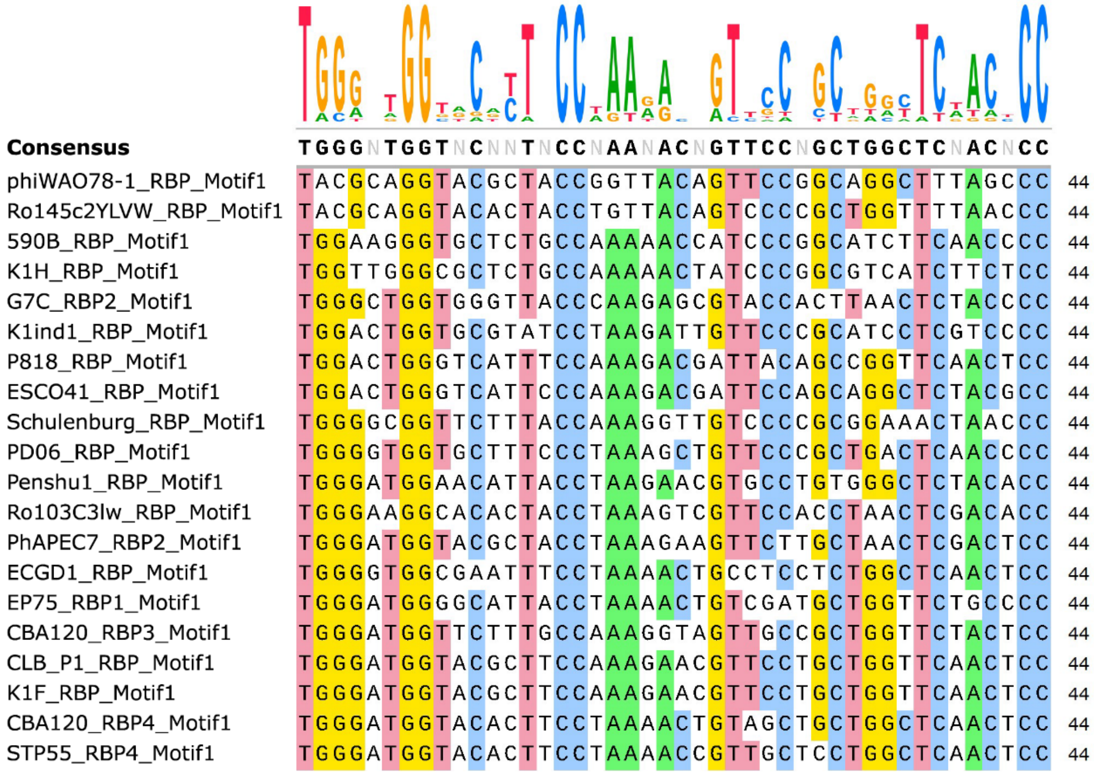

Additional file 6: Figure S6: Multiple sequence alignment of motif one. This 44 nt long motif DNA sequence is located at the end of the anchor domain of the RBP.

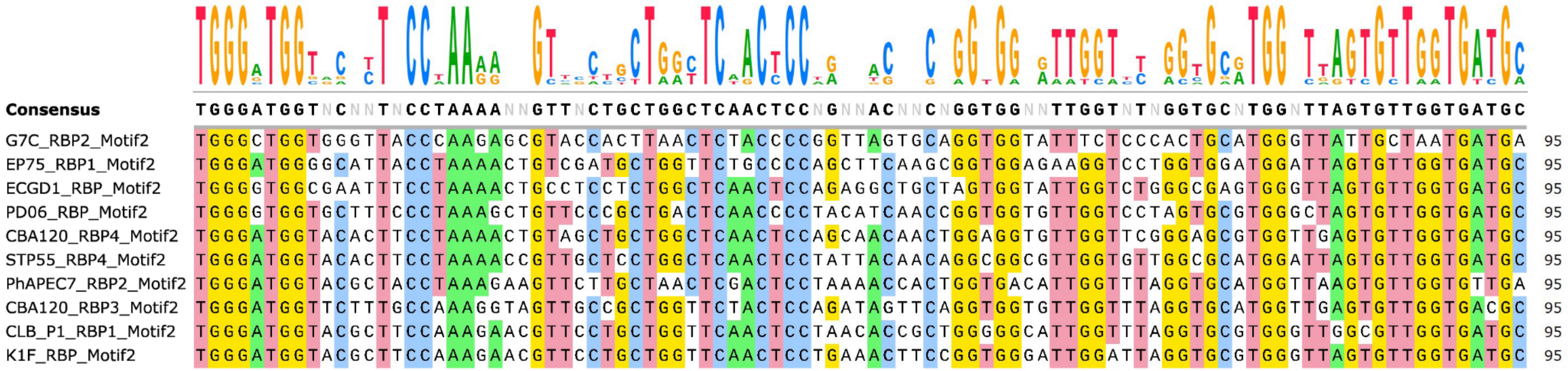

Additional file 6: Figure S7: **Multiple sequence alignment of motif two.** The 95 nt long region is an extended version of motif 1 and is located at the end of the anchor domain of the RBP.

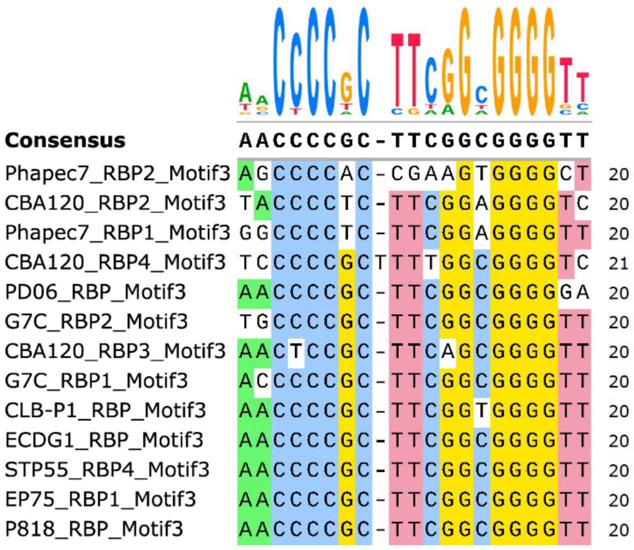

Additional file 6: Figure S8: **Multiple sequence alignment of motif three.** This 20 nt long region is located in the noncoding DNA sequence downstream of the RBP coding sequence. The sequence starts between zero and nineteen nucleotides downstream of the stop codon, and is also predicted to function as a transcription terminator.
